# Increased GABA Contributes to Enhanced Control over Motor Excitability in Tourette Syndrome

**DOI:** 10.1016/j.cub.2014.08.038

**Published:** 2014-10-06

**Authors:** Amelia Draper, Mary C. Stephenson, Georgina M. Jackson, Sophia Pépés, Paul S. Morgan, Peter G. Morris, Stephen R. Jackson

**Affiliations:** 1 School of Psychology, University of Nottingham, Nottingham NG7 2RD, UK; 2 Sir Peter Mansfield Magnetic Resonance Centre, University of Nottingham, Nottingham NG7 2RD, UK; 3 Division of Psychiatry and Applied Psychology, Institute of Mental Health, School of Medicine, University of Nottingham, Nottingham NG7 2TU, UK; 4 Medical Physics and Clinical Engineering, Queen’s Medical Centre, Nottingham NG7 2RD, UK

## Abstract

Tourette syndrome (TS) is a developmental neurological disorder characterized by vocal and motor tics [1] and associated with cortical-striatal-thalamic-cortical circuit dysfunction [2, 3], hyperexcitability within cortical motor areas [4], and altered intracortical inhibition [4, 5, 6, 7]. TS often follows a developmental time course in which tics become increasingly more controlled during adolescence in many individuals [1], who exhibit enhanced control over their volitional movements [8, 9, 10, 11]. Importantly, control over motor outputs appears to be brought about by a reduction in the gain of motor excitability [6, 7, 12, 13]. Here we present a neurochemical basis for a localized gain control mechanism. We used ultra-high-field (7 T) magnetic resonance spectroscopy to investigate in vivo concentrations of γ-aminobutyric acid (GABA) within primary and secondary motor areas of individuals with TS. We demonstrate that GABA concentrations within the supplementary motor area (SMA)—a region strongly associated with the genesis of motor tics in TS [14]—are paradoxically elevated in individuals with TS and inversely related to fMRI blood oxygen level-dependent activation. By contrast, GABA concentrations in control sites do not differ from those of a matched control group. Importantly, we also show that GABA concentrations within the SMA are inversely correlated with cortical excitability in primary motor cortex and are predicted by motor tic severity and white-matter microstructure (FA) within a region of the corpus callosum that projects to the SMA within each hemisphere. Based upon these findings, we propose that extrasynaptic GABA contributes to a form of control, based upon localized tonic inhibition within the SMA, that may lead to the suppression of tics.

## Highlights

• We report a 7 T ^1^H MRS investigation of GABA in Tourette syndrome (TS)
• GABA levels within the SMA are significantly elevated in TS
• SMA GABA is negatively correlated with SMA BOLD and cortical excitability
• SMA GABA is predicted by motor tic severity and corpus callosum FA values

## Teaser

Using magnetic resonance spectroscopy, Draper et al. find that concentrations of the neurotransmitter GABA in motor areas of the brain are associated with symptom severity in individuals with Tourette syndrome.

## Results

Tourette syndrome (TS) is associated with alterations in the development of brain networks that result in neural circuits with imbalanced excitatory and inhibitory influences [15]. It is generally acknowledged that cortical-striatal-thalamic-cortical (CSTC) circuits are dysfunctional in TS, with subsets of striatal neurons becoming active within inappropriate contexts, resulting in the disinhibition of thalamocortical circuits [3] and the hyperexcitability of motor regions of the brain [4, 5, 6, 7] that in turn lead to the occurrence of tics [14].

TS has been linked to alterations in inhibitory γ-aminobutyric acid (GABA) signaling [15, 16]. Postmortem examination has demonstrated that there are substantial decreases in the number of GABA interneurons found within the striatum of individuals with TS [2], and positron emission tomography imaging has revealed widespread reductions in GABA_A_ receptor binding in TS [17]. Finally, studies of cortical-spinal excitability (CSE) in TS have demonstrated reduced intracortical GABAergic inhibition [4, 5, 6, 7]. Together, these findings predict reduced phasic GABAergic inhibition in individuals with TS, which has most often been interpreted as a primary cause of the disorder contributing to the occurrence of tics.

TS often follows a developmental time course characterized by a reduction in the frequency and intensity of tics during adolescence [1]. It has been proposed that individuals gain control over their tics through the development of compensatory mechanisms that lead to enhanced control over motor outputs based upon increased tonic inhibition [8, 10, 11, 18, 19, 20]. Consistent with this proposal, it has been shown that the gain of transcranial magnetic stimulation (TMS)-induced motor excitability (i.e., TMS recruitment curves) ([6]; see Supplemental Information available online) and the gain of motor excitability immediately prior to volitional movements are both significantly reduced in individuals with TS [7, 12, 13]. These findings have been interpreted as a secondary consequence of, or adaptation to, the disorder and have been associated with a reduction in clinical symptoms [6]. Importantly, it has been demonstrated that both reduced inhibition (e.g., reduced short-interval cortical inhibition) and enhanced inhibition (e.g., reduced gain for TMS-induced motor excitability) are observed in the same group of individuals with TS [6].

The supplementary motor area (SMA) is a likely focus for these control mechanisms. The SMA is a major site for thalamocortical projections [21] and has been linked previously to the volitional control of action [22] and nonconscious, effector-specific control of motor outputs [23]. GABA concentrations within the SMA are correlated with performance on behavioral tasks that index nonconscious control of motor outputs [24]. Most importantly, cortical excitability within the SMA is linked to the genesis of tics in TS. Thus, the hyperexcitability within primary motor cortex (M1) that is observed in TS is likely due to increased functional interaction between SMA and M1 [25]: there is increased activity in the SMA of individuals with TS that immediately precedes the occurrence of tics [14], and inhibitory repetitive TMS (rTMS) delivered to the SMA has been shown to decrease tic frequency in individuals with TS [26, 27, 28]. Here we offer a novel perspective on how this increased control over motor outputs can arise as a consequence of localized increases in tonic inhibition based upon increased levels of nonsynaptic, extracellular GABA concentration that operate to alter the gain of cortical spinal excitability (CSE) locally.

**Figure 1:**
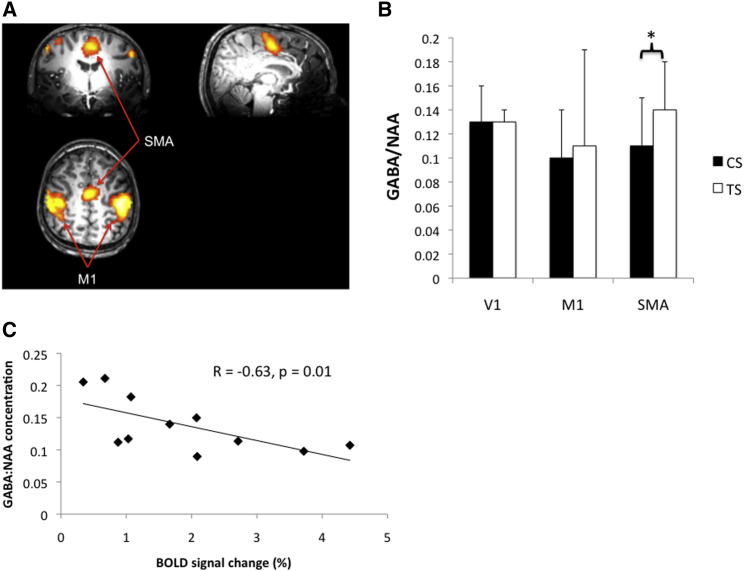
Results of Brain Imaging Analyses (A) The fMRI BOLD signal associated with a bimanual sequential finger-thumb opposition task (tap > rest contrast) for a single representative participant that, for presentation purposes only, has been spatially smoothed. (B) Mean GABA/NAA ratios for the Tourette syndrome (TS) and control group (CS) for each ROI. For the SMA ROI only, GABA concentrations are elevated relative to controls (^∗^p < 0.05). Error bars are standard deviation. (C) Scatterplot showing the negative association (r = −0.63, p < 0.01) between individual GABA/NAA ratios in the SMA voxel and fMRI BOLD signal change values within that same voxel.

We used ^1^H magnetic resonance spectroscopy (MRS) at ultra-high field (7 T) to investigate in vivo concentrations of GABA within the primary and secondary (SMA) motor areas of 15 adolescents (mean age 15.75 ± 3.05 years) with a confirmed clinical diagnosis of TS and a control group of age- and gender-matched typically developing individuals. MR spectroscopy data were collected from three 20 mm^3^ regions of interest (ROIs) located within the hand area of left primary sensorimotor cortex (M1), and bilaterally from within the SMA and the primary visual cortex (V1). To aid localization of the hand area of M1 and SMA, participants performed a brief bimanual, sequential finger-thumb opposition task (i.e., with both hands continuously tap each finger sequentially against the thumb until instructed to stop) while functional MR images were obtained (Figure 1A).

### Tissue Fraction Analyses

Anatomical (MP RAGE) images were analyzed to estimate, for each participant, the fraction of cerebral spinal fluid (CSF), gray matter (GM), and white matter (WM) within each volume of interest (VOI). The CSF, GM, and WM fractions were compared separately between groups using independent-samples t tests. These analyses confirmed that there were no significant differences in the proportion of each tissue type between groups for each VOI (maximum t(27) = 1.2, p > 0.1).

### fMRI BOLD Analyses

Group differences in the magnitude of the fMRI blood oxygen level-dependent (BOLD) signal change for the tap > rest behavioral contrast were examined for each of the M1 and SMA VOIs using an independent-samples t test. The TS group had a significantly larger BOLD signal within the SMA VOI compared to controls (means: TS group = 1.88% ± 1.3%, control group = 1.1% ± 0.8%; t(27) = 2.1, p < 0.05). There was no significant between-group difference in fMRI BOLD within the M1 voxel.

### GABA

The TS group had significantly increased absolute concentrations of GABA within the SMA compared to the control group (means: TS group = 1.1 ± 0.43 μmol/g, control group = 0.75 ± 0.28 μmol/g; t(27) = 2.5, p < 0.05). GABA concentrations within the M1 and V1 ROIs did not differ significantly between groups (both p > 0.5).

### GABA/NAA Ratio

Following convention (e.g., [29]), we measured GABA concentrations as a ratio of *N*-acetylaspartate (NAA) concentrations within each ROI. Importantly, preliminary analyses confirmed that NAA concentrations did not differ significantly between groups for any of the three ROIs. The analyses revealed that GABA/NAA ratios were significantly increased relative to the matched control group within the SMA (means: TS group = 0.14 ± 0.04, control group = 0.11 ± 0.04; t(27) = 2.2, p < 0.05) but were not different from control group levels for the M1 or V1 ROIs (Figure 1B). (See Supplemental Information for identical findings for the GABA/creatine ratio.)

Our finding of selectively increased concentrations of GABA within the SMA in individuals with TS (hereafter MRS-GABA) is consistent with the proposal that MRS-GABA primarily measures extracellular GABA concentrations that have been linked to alterations in levels of tonic inhibition ([30]; see [31] for review). A discussion of the cellular basis for tonic inhibition is beyond the remit of this paper (but for recent reviews see [32, 33, 34, 35]).

It should be noted that MRS-GABA was measured at rest in the current study, and participants were instructed to remain still throughout. This would require individuals with TS to actively suppress their tics. We cannot rule out that active suppression of tics contributed to the increased MRS-GABA in SMA that we observed. An increase in MRS-GABA in the SMA during tic suppression in TS would be consistent with previous reports that MRS-GABA concentrations within the SMA of neurologically healthy individuals correlate with individual levels of performance on behavioral tasks that index control of motor outputs [24].

### Relationship between GABA Concentration and fMRI BOLD Response in TS

We investigated the association between MRS-GABA and fMRI BOLD within the SMA and M1 voxels. Previous studies reported a negative correlation between MRS-GABA concentrations and fMRI BOLD in the visual and motor cortices of healthy adults [29, 36, 37]. Pearson correlation coefficients were calculated for each group separately for each VOI. For the TS group, the analyses confirmed that the fMRI BOLD signal change within the SMA voxel was significantly negatively correlated with GABA/NAA ratio (r = −0.65, p < 0.01). Scatterplots showing these data are presented in Figure 1C. The correlation between fMRI BOLD response and GABA/NAA within the M1 VOI did not approach statistical significance (p > 0.1). The correlations between MRS-GABA and fMRI BOLD in M1 and SMA did not reach statistical significance for controls. Our finding that MRS-GABA concentrations within the SMA are inversely associated with the fMRI BOLD response is consistent with previous reports [29, 36, 37]. It is also consistent with the proposal that increases in MRS-GABA are linked to localized increases in tonic inhibition [30, 31], and that increased control over motor outputs in TS are brought about by reducing the gain of corticospinal excitability in cortical motor regions through increased tonic inhibition [7, 12, 13].

### Relationship between GABA Concentration and Cortical-Spinal Excitability in TS

Individuals with TS exhibit significantly reduced gain for TMS-induced CSE [6] and also preceding the execution of volitional movements [7, 12, 13]. Furthermore, gain in CSE is inversely related to tic severity ([13]; see Supplemental Information). These findings have been interpreted as evidence that the gain of CSE is reduced in TS due to increased levels of tonic inhibition [7, 13].

**Figure 2:**
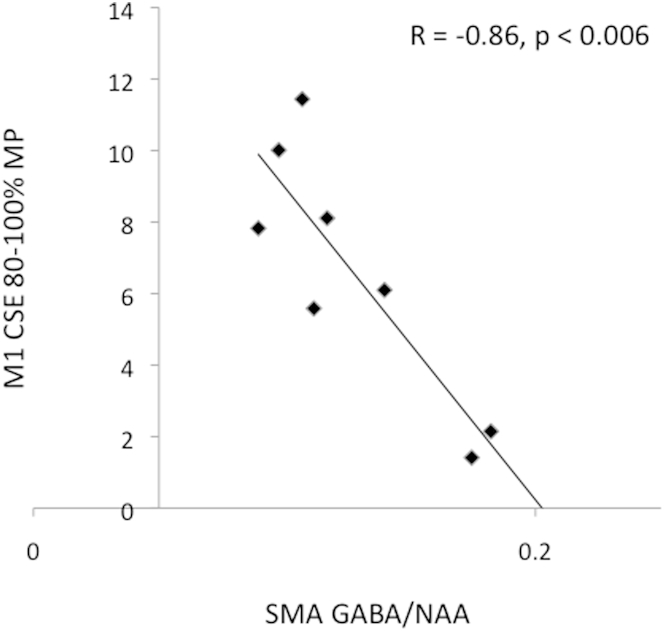
Association between SMA GABA Concentration and Motor Excitability Individual levels of GABA concentration within the SMA ROI are inversely related to motor excitability immediately prior (81%–100%) to the execution of volitional movements. Motor excitability was measured by recording motor evoked potentials from the right hand following single-pulse TMS delivered to the left primary motor cortex. See Supplemental Information for further details.

To investigate the relationship between MRS-GABA and CSE in TS, we examined the Pearson correlation between MRS-GABA within SMA and levels of CSE within primary motor cortex (M1). CSE was measured using single-pulse TMS delivered to the hand area of the left M1 region in the period immediately preceding (81%–100% of the movement preparation period) volitional movements of the right hand in a subset of TS patients who had taken part in the current study and also in the study reported by Draper et al. [13]. This analysis revealed a significant negative correlation (R = −0.86, p < 0.006) between MRS-GABA in the SMA ROI and CSE measured within the left M1 (Figure 2). Although statistically significant, this result should be interpreted with caution due to the relatively small sample size. Nevertheless, taken together with the finding for fMRI BOLD reported above, it indicates that increases in MRS-GABA within the SMA in the TS group are likely associated with decreases in motor excitability.

If MRS primarily measures nonsynaptic, extracellular GABA concentrations that are linked to ambient levels of tonic inhibition [30, 31], then a key issue is to understand factors associated with MRS-GABA increases. One possibility is that MRS-GABA increases are triggered by neural projections arriving from other brain areas; another is that they are associated with tic severity scores.

### Relationship between SMA GABA Concentration and WM Microstructure within the Corpus Callosum

As noted previously, the SMA is a major site for thalamocortical and cortical-cortical projections [21], and cortical excitability within the SMA is strongly linked to the genesis of tics in TS. Previous studies have demonstrated altered WM microstructure (i.e., reduced fractional anisotropy [FA] values in TS relative to matched controls) within regions of the corpus callosum (CC) linking sensorimotor areas of cortex. Furthermore, these alterations in FA are positively correlated with tic severity [20, 38].

**Figure 3:**
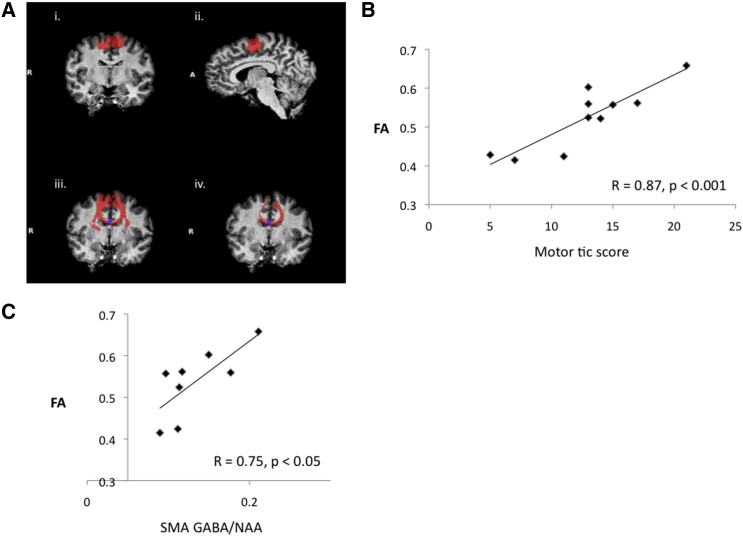
Association between SMA GABA Concentration and WM Microstructure (Ai and Aii) An example ROI located in the SMA, displayed in the axial (Ai) and sagittal (Aii) planes. (Aiii) Fibers tracked from the SMA region (red) and the location of the 6 mm^3^ ROI located within the body of the corpus callosum where the fibers cross between the hemispheres (blue). (Aiv) Confirmation that fibers tracked from the central 6 mm^3^ ROI located within the corpus callosum terminate within the SMA. (B) Scatterplot showing the significant positive association (r = 0.87, p < 0.001) between individual fractional anisotropy (FA) values within corpus callosum ROI and motor tic severity. (C) Scatterplot showing the significant positive association (r = 0.75, p < 0.05) between individual FA values within corpus callosum ROI and GABA concentration values within the SMA voxel.

We used diffusion tensor imaging (DTI) and tractography to test the hypothesis that increased projections to and from the SMA, as measured by FA values in the region of the CC projecting to the SMA, would be positively associated in TS with increased tic severity and increased MRS-GABA in the SMA. We identified a 6 mm^3^ ROI within the body of the CC from which fibers clearly connected to the SMA within each hemisphere (Figure 3A; see Supplemental Information for details). We then measured mean FA values within this CC ROI for the TS group and correlated these with tic severity scores and MRS-GABA within the SMA ROI. The analyses revealed that FA values within the CC ROI were positively correlated with motor tic severity scores (R = 0.87, p < 0.001). These data confirm the previous finding that a reduction in CC projections to motor cortical areas is associated with reduced motor tic severity [20, 38]. More importantly, the analyses also revealed that FA within the CC ROI was significantly positively correlated with MRS-GABA within the SMA (R = 0.75, p < 0.05).

### Linear Regression Analyses

To investigate which of several key variables might contribute to MRS-GABA concentrations within the SMA in the TS group, we carried out linear regression analyses for the following variables: participant age, current tic severity (Yale Global Tic Severity Scale [YGTSS] impairment, global, and motor scores), and WM microstructure (FA) in the region of the CC projecting to the SMA. The analyses confirmed that participant age and nonmotor indices of tic severity (i.e., YGTSS impairment and global scores) were not significant predictors of MRS-GABA within the SMA (p > 0.05). By contrast, motor tic severity (YGTSS motor score) (RSq = 0.54, Adj-Rsq = 0.46, F = 6.97, p < 0.04) and callosal FA (RSq = 0.62, Adj-Rsq = 0.55, F = 9.7, p < 0.025) were each significant predictors of MRS-GABA within the SMA for the TS group.

However, callosal FA is itself highly positively correlated with motor tic severity (R = 0.87, p < 0.001). To determine the joint contribution of these factors, we entered them into a stepwise regression model. The analyses demonstrated that when motor tic severity is entered into the model first, callosal FA accounts for no additional variance and is no longer a significant predictor of MRS-GABA (t = 1.13, p > 0.1). By contrast, if callosal FA values are entered into the model first, motor tic severity accounts for no additional variance and is no longer a significant predictor of SMA GABA (t = 0.42, p > 0.1).

## Discussion

We used ultra-high-field (7 T) ^1^H MRS to investigate for the first time in vivo concentrations of GABA within primary and secondary motor areas of individuals with Tourette syndrome (TS). We demonstrate that concentrations of GABA within the SMA—a brain area consistently linked with the cortical genesis of motor tics in TS—are significantly elevated in individuals with TS relative to a control group of age-matched typically developing individuals. By contrast, GABA levels in primary motor cortex (M1) and in a control site within occipital cortex (V1) do not differ between groups.

We investigated the relationship between elevated MRS-GABA observed for the TS group and measures of motor tic severity, motor cortical excitability, fMRI BOLD response, and the structural connectivity of the SMA region. We demonstrate that MRS-GABA within the SMA is strongly negatively correlated with the fMRI BOLD response in SMA and also cortical excitability values within sensorimotor cortex, as measured by single-pulse TMS delivered immediately prior to a volitional movement of the contralateral hand [13]. Importantly, we report for the first time that MRS-GABA levels within the SMA are strongly positively predicted by both motor tic severity and the FA values within a region of the CC that projects to the SMA, and that these factors are themselves highly positively correlated.

It has been argued that an important secondary consequence of TS is that enhanced control over volitional movements, and the suppression of tics, may arise as a result of increased tonic inhibition [7, 8, 9, 10, 11, 12, 13]. This proposal is consistent with the repeated finding that the gain in cortical excitability is reduced in TS ahead of volitional movements [7, 12, 13] and in response to increasing levels of TMS stimulation [6]. Based upon the findings of the current study, we propose that this increase in tonic inhibition may be due to localized increases in extracellular GABA within the SMA. We believe that these findings are particularly important for understanding how localized adaptive changes in brain function may accompany neurodevelopmental disorders and play a key role in the control of behavioral symptoms.

## Supplemental Information

Document S1. Figures S1–S3, Tables S1 and S2, and Supplemental Experimental Procedures

Document S2. Article plus Supplemental Information
