# Supplementary material for: Increased GABA Contributes to Enhanced Control over Motor Excitability in Tourette Syndrome
Source: Curr Biol. 2014 Oct 6;24(19):2343–7. doi: 10.1016/j.cub.2014.08.038 (PMC4188813; doi:10.1016/j.cub.2014.08.038)
Supplement: Document S1. Figures S1–S3, Tables S1 and S2, and Supplemental Experimental Procedures [file mmc1.pdf]

Current Biology, Volume *24*

Supplemental Information

Increased GABA Contributes to Enhanced Control over Motor Excitability in Tourette Syndrome

Amelia Draper, Mary C. Stephenson, Georgina M. Jackson, Sophia Pépés, Paul S. Morgan, Peter G. Morris, and Stephen R. Jackson

Supplemental Information

Supplemental Figures

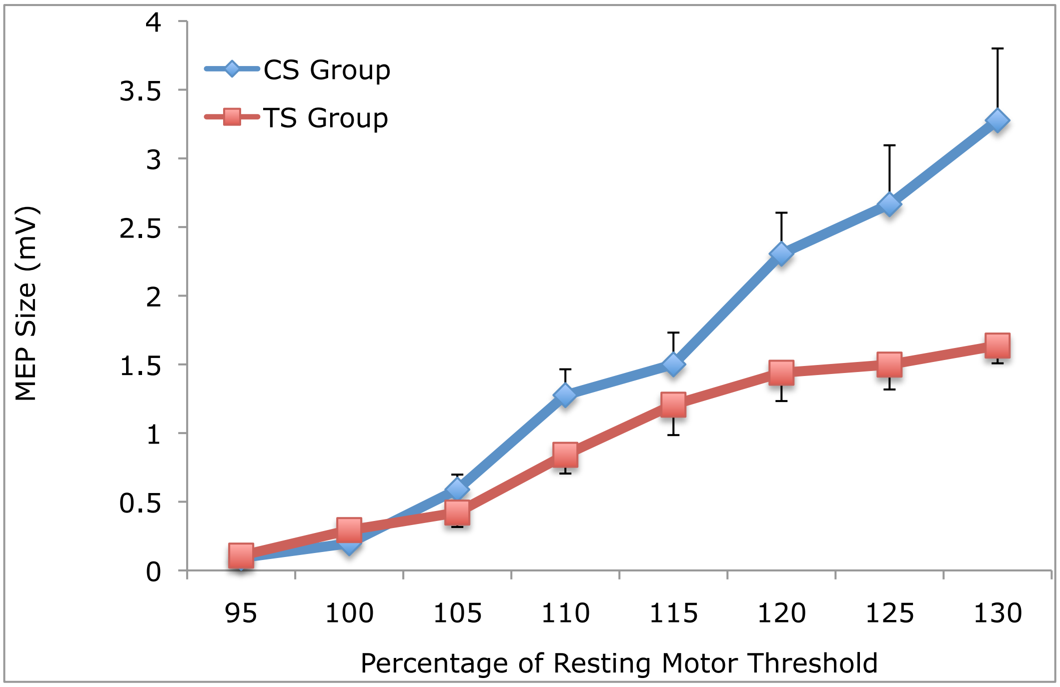

Figure S1: This figure relates to Figure 2 of the main text. It illustrates the recruitment curves for TMS-induced cortical spinal excitability for the TS group and controls. The graph shows the average MEP value for each group obtained from the FDI muscle of the right hand following single pulse TMS delivered to the left hand motor area at 95% - 130% of Resting Motor Threshold. Error bars are Standard Error.

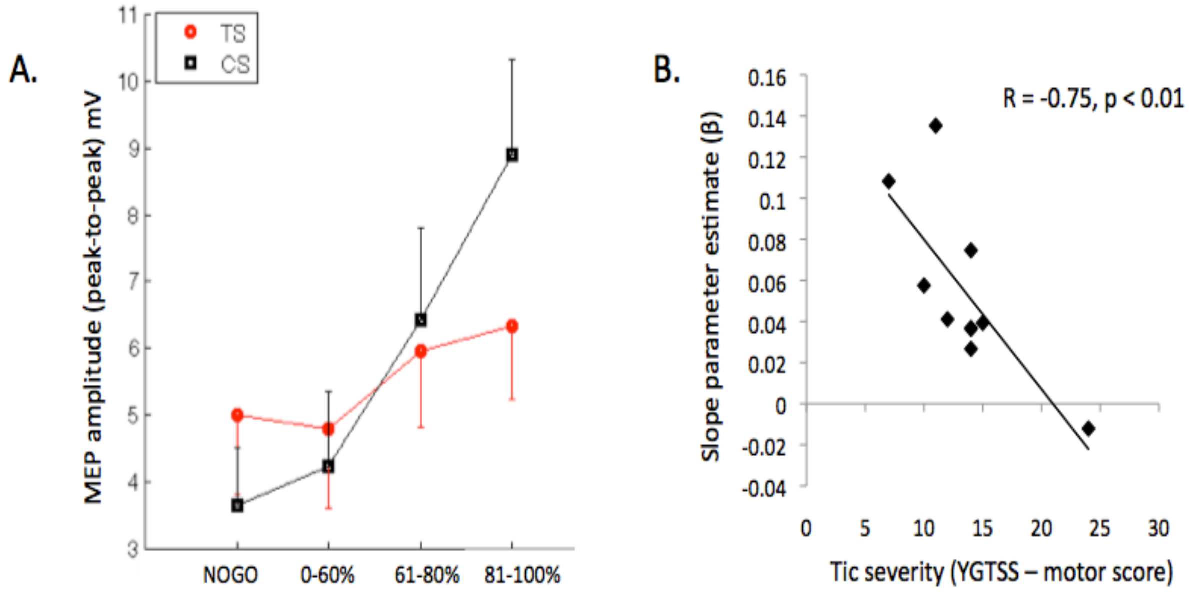

Figure S2: A. Data reproduced from Draper et al. [**S3**]. Illustrates differences between TS patients and matched controls in motor excitability immediately preceding the execution of volitional movements in a Go/NoGo task. The figure shows mean TMS-induced MEP amplitudes for TS and control groups when single pulse TMS was delivered on NoGo trials and 0-60%, 61-80%, or 81-100% of the movement preparation period on Go trials. Error bars are the standard error of the mean. **B.** Data reproduced from Draper et al. [**S3**] illustrating the relationship between motor tic increases in motor excitability, as indexed by the rise in MEP amplitude as a function of time through the movement preparation period.

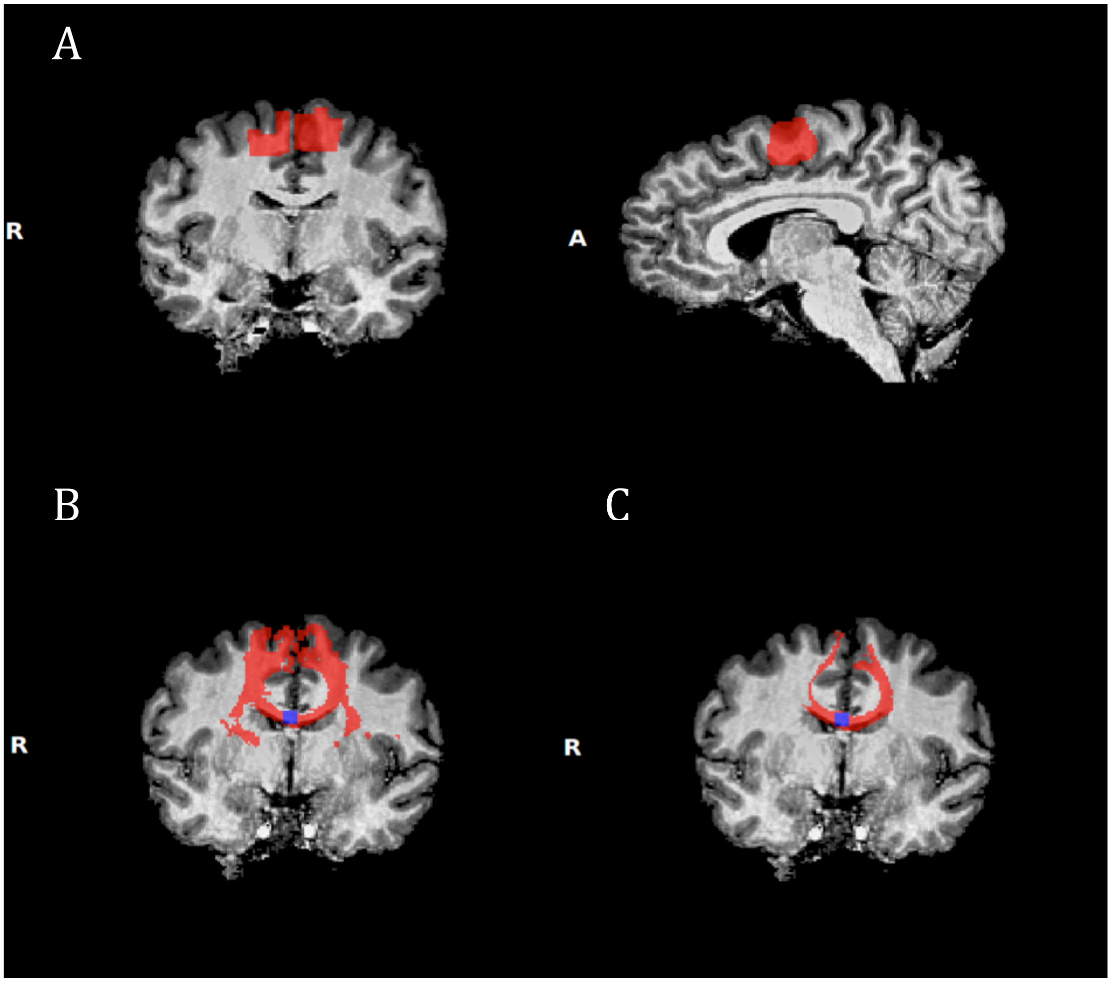

Figure S3: This figure relates to Figure 3 of the main text. A: Example of an ROI located in the supplementary motor area in the axial and sagittal plane. B: Illustrates fibres tracked from the SMA (red) and also the location of the $6mm^3$ ROI positioned within the body of the corpus callosum where the fibres cross between the cerebral hemispheres (blue). C: Illustrates fibres that were tracked from the central $6mm^3$ ROI terminate in the SMA.

Supplemental Tables

The focus of the current study was to investigate differences between the TS group and matched controls in MRS-GABA concentration within the primary and secondary motor areas, however for completeness we also report between group comparison of some other metabolites or metabolite ratios that may be of interest. These data are presented in Table S1 below.

N-acetylaspartate [NAA]

Absolute and tissue-corrected NAA concentration (i.e., absolute NAA values corrected for individual GM fractions) did not differ between the groups within any of the three ROIs.

GABA:NAA and GABA:Creatine (Cre) ratios

As reported in the main text, the GABA:NAA ratio was significantly increased for the TS group, but only within the SMA ROI. While we prefer to report the GABA:NAA ratio in the main text so as to maintain consistency with previous studies reporting GABA:NAA ratios, it is important to note that this finding is also observed if we examine for the GABA:Cr ratio (Table S1).

GABA/Glutamate (Glu) ratio

Effective cortical processing requires balanced excitatory and inhibitory influences. Glutamate is the main excitatory neurotransmitter within the human brain and GABA the main inhibitory neurotransmitter. As TS has been linked previously to imbalances in ambient levels of these neurotransmitters, we compared directly the GABA/Glu ratio for each group. The TS group had significantly higher GABA/Glu ratios in the SMA compared to those of the matched control group ($p<0.009$). By contrast, GABA/Glu ratios did not differ significantly between groups for either the M1 or V1 voxels (Table S1).

Glutamine (Gln)/Glu ratio

Within the brain the glutamine-glutamate cycle is key to the turnover and synthesis of both glutamate and GABA [**S1**]. Glutamate removed from the synaptic cleft is converted to glutamine within astrocytes. Astrocyte-derived glutamine is then used as a precursor for the synthesis of glutamate or GABA within neurons. For this reason, the Gln/Glu ratio is viewed as a potential marker for increases in brain activity. In the current study we found a marginal increase in the Gln/Glu ratio for the TS group within the SMA ROI ($p = 0.06$). The Gln/Glu ratio did not differ significantly between groups for either the M1 or V1 voxels.

**SMA ROI**			
**Metabolite**	**CS group**	**TS group**	**p-value**
*NAA**	6.9 ±0.47	7.3 ±1.27	NS
NAA	16.4 ±8.4	16.3 ±7.6	NS
GABA:NAA	0.11 ±0.04	0.16 ±0.06	**0.007**
GABA:Cr	0.26 ±0.14	0.34 ±0.14	**0.04**
GABA:Glu	0.10 ±0.04	0.13 ±0.04	**0.009**
Gln:Glu	0.23 ±0.07	0.26 ±0.08	0.06
**M1 ROI**			
**Metabolite**	**CS group**	**TS group**	**p-value**
*NAA**	8.0 ±0.82	7.3 ±1.25	NS
NAA	28.0 ±8.2	27.1 ±11.6	NS
GABA:NAA	0.10 ±0.04	0.11 ±0.08	NS
GABA:Cr	0.27 ±0.08	0.27 ±0.16	NS
GABA:Glu	0.11 ±0.04	0.10 ±0.05	NS
Gln:Glu	0.19 ±0.05	0.22 ±0.1	NS
**V1 ROI**			
**Metabolite**	**CS group**	**TS group**	**p-value**
*NAA**	8.9 ±0.8	8.4 ±0.8	NS
NAA	23.5 ±8.2	18.9 ±6.8	NS
GABA:NAA	0.13 ±0.03	0.13 ±0.06	NS
GABA:Cr	0.35 ±0.12	0.40 ±0.19	NS
GABA:Glu	0.13 ±0.03	0.14 ±0.06	NS
Gln:Glu	0.23 ±0.06	0.22 ±0.10	NS

Table S1: Comparison of additional metabolite concentrations and metabolite ratios for each group and region-of-interest (ROI). Absolute metabolite concentrations were standardised for each participant using that individual's measured GM fraction for each ROI. For completeness, and to aid comparison with previous studies, uncorrected concentrations of NAA are also presented (NAA*).

ID	Age (years)	Gender	IQ (WASI)	YGSS	Motor tic scores	Phonic tic scores	Co-morbitity	Medication
TS006	20.0	M	84	50	14	16	-	Clonidine
TS018	18.2	M	120	41	13	5	ADD	Clonidine
TS028	17.8	F	96	41	10	15	OCD	-
TS071	12.8	M	118	83	22	11	OCD	-
TS013	17.2	M	135	47	11	6	OCD	-
TS043	11.5	M	123	67	17	10	-	Clonidine
TS069	11.7	M	133	20	10	0	-	-
TS034	13.8	M	118	No tics last 2 months			-	-
TS062	17.0	M	131	65	24	21	-	Citalopram
TS048	14.8	M	118	28	12	6	-	Clonidine
TS049	20.2	M	116	64	21	13	-	Kapra
TS007	19.7	M	95	25	5	0	-	Clonidine
TS030	15.0	M	103	61	16	15	-	-
TS076	14.2	M	85	40	13	8	ADHD	Methylphenidate, Melatonin
TS074	12.4	M	102	30	10	8	-	Risperidone

Table S2: Clinical characteristics of subjects with Tourette's Syndrome (note: YGSS = Yale Global Tic Severity Scale; WASI= Wechsler Abbreviated Scale of Intelligence: vocabulary and matrix reasoning subtests; ADD = Attention Deficit Disorder; OCD = Obsessive-Compulsive Disorder; ADHD = Attention Deficit Hyperactivity Disorder).

Supplemental Experimental Procedures

1. Investigation of TMS-induced modulation of cortical-spinal excitability

Participants

Seventeen participants in total took part in this TMS study. Eight participants had Tourette syndrome, including 6 males, with a mean age of 18.3 ±2.7 years and a total Yale tic score ranging from 3 to 51. Nine controls were recruited that were gender and age matched to the TS group (7 males, mean age of 17.2 ±3.3 years).

Procedures

An unpaired Magstim Bistim 2 machine together with a 70mm figure-of-eight coil was used to deliver single-pulse TMS to the motor hotspot of the first dorsal interroseous (FDI) muscle in the right hand.

The motor hotspot was determined by a trial-and-error procedure. Once it had been found the resting motor threshold (RMT) was estimated at the motor hotspot based upon the stimulator intensity required to elicit an MEP of at least 150-200μV in 5 out of 10 trials. The location of this motor hotspot was subsequently tracked throughout the experiment using BrainSight 2. Specifically, a BrainSight tracking device was attached to the participant's forehead and to the TMS coil. A camera and software that aligns specific points on the participant's head to a virtual head display, and automatic curvilinear registration, allowed the experimenter to ensure that the TMS coil was always placed over the motor hotspot.

Once the motor hotspot was located, the TMS coil was stabilised using a manfrotto arm. The coil was continuously observed by the experimenter and adjusted whilst the trials were delivered to ensure the coil was always correctly positioned over the target. In order to record the TMS-induced muscle response, disposable electromyography electrodes with a diameter of 5mm were placed on the FDI muscle in a standard belly-tendon configuration. BrainVision Recorder software was used to record responses to the TMS protocol.

Participants rested their chin on a chin-rest whilst receiving 80 trials of single pulse TMS delivered at different percentages of their RMT with 5 seconds in between each

pulse. Intensities were delivered from 95% - 130% of RMT in increments of 5%. Pulses were pseudo-randomised and organised into 8 blocks (i.e., each block contained one trial of each intensity in a random order). After each block the experimenter checked that the participant was tolerating the procedure well and could readjust the coil position as necessary.

Results

Recruitment curves for the TS and control groups are presented in Figure S1. Inspection of this figure clearly indicates that the gain in motor excitability, as measured by mean MEP from the FDI muscle following single pulse TMS delivered to the left hand motor area at 95% - 130% of RMT, is reduced in the TS group.

To investigate this effect we carried out a 2-way mixed ANOVA with the between-subject factor Group (CS vs. TS) and the within subject factor Stimulator output (95% - 130% RMT). This analysis revealed significant main effects of Group ($F(1,15) = 6.2$, $p = 0.025$) and Stimulator output ($F(7,105) = 44.6$, $p < 0.00001$), and a significant Group x Stimulator output interaction ($F(7,105) = 5.4$, $p < 0.0001$).

This result confirms previous reports [**S2**] that the gain of TMS-induced motor excitability is significantly reduced in TS.

2. Cortical-spinal excitability prior to volitional movement in TS

A study investigating alterations in motor cortical-spinal excitability (CSE) ahead of volitional movements in individuals with TS, that was reported by Draper et al., [**S3**], was conducted concurrently with the current study, and eight of the current participants participated in both studies. As the Draper et al. study [**S3**] used TMS techniques to measure directly CSE ahead of volitional movements, it provides additional, independent, converging, evidence with respect to the relationship between MRS-GABA and motor excitability, and thus supports the finding reported in the current study that individual MRS-GABA concentrations are inversely related to fMRI BOLD activations. Full methodological details of the Draper et al., study are provided in [**S3**] and are summarised below. Figure S2 illustrates the core findings from the Draper et al. study.

Transcranical Magnetic Stimulation

Single pulse TMS was delivered to the hand area of the left hemisphere motor cortex. The TMS coil was positioned over the motor hotspot for the first dorsal interosseous (FDI) muscle of the right hand. Resting motor threshold (RMT) was estimated using an adapted staircase procedure and TMS pulse intensity was set to 110% of RMT throughout the experiment. Motor-evoked potentials (MEPs) were obtained from the FDI muscle of the right hand on each trial.

Experimental Procedure

Participants were seated with their head resting comfortably in a chin rest positioned 50cm away from a 17inch monitor that displayed the stimuli and completed a GO/NOGO motor task. On GO trials subjects pressed a response button with their right index finger whenever a 4cm diameter green filled circle, located centrally on a grey background, was presented to them. A red filled circle indicated a NOGO trial and subjects were instructed to withhold a response whenever the red circles appeared. Trials were presented in a pseudo-random order.

Participants completed 120 experimental trials and their median reaction time was re-calculated every 6 trials to control for any change in average speed of responding. A single TMS pulse was delivered on all trials at 25%, 50% or 75% of the individual's median reaction time relative to stimulus onset.

Electromyography (EMG) data was recorded on each trial for a period of 3 seconds following stimulus onset. EMG signals buffered and amplified using a g.USB Biosignal Amplifier (g-tech) with a sampling frequency of 1200Hz.

Statistical analyses

MEPs for all trials were visually inspected and any trials in which the MEP was ambiguous were excluded. TMS-induced MEPs were identified as the signal occurring directly after the characteristic TMS artefact, and MEP amplitude was defined as the peak-to-peak difference in amplitude measured in millivolts (mV). The *response-locked*

timing for when TMS pulse was delivered during the pre-movement period was calculated for each GO trial and expressed as the percentage of the reaction time for that trial, calculated as the interval separating stimulus onset time (0%) and response time (100%). Data were separated for each participant for TMS pulses delivered at 81-100% of *movement preparation* time. Median MEP amplitudes were then calculated for each individual.

3. ^{1}H Magnetic resonance Spectroscopy study

<u>Participants</u>

Fifteen adolescents (mean age = 15.75 ± 3.05 years, range = 11-20 years) with a confirmed clinical diagnosis of Tourette syndrome (TS) took part in this study. Participants were recruited from a specialised Tourette syndrome clinic at Queen's Medical Centre, Nottingham. Fourteen age and gender-matched (1 female, 13 males, mean age 15.86 ± 3.24 years) typically developing adolescents were recruited to act as a control group. Tic symptoms were measured on the day of testing using the Yale Global Tic Severity Scale (YGTSS) [**S4**]. Three TS participants had an additional diagnosis of co-morbid Obsessive Compulsive Disorder (OCD), one had an additional diagnosis of Attention Deficit Disorder (ADD) and one an additional diagnosis of Attention Deficit Hyperactivity Disorder (ADHD). Details of TS participants are presented in Table S2. For both groups IQ was estimated using the Wechsler Abbreviated Scale of Intelligence (WASI) using only the vocabulary and matrix reasoning subtests. Independent t-tests established that there were no significant differences between the groups in Age (TS group mean=15.75, CS group mean= 15.86, $t(27) = -0.1$, $p>0.1$) or IQ (TS group mean = 113, CS group mean = 120, $t(27) = 1.2$, $p>0.1$).

<u>Magnetic Resonance Data Acquisition</u>

Magnetic resonance data were acquired on a 7 Tesla Philips Achieva magnetic resonance imaging scanner with a 32-channel SENSE radio-frequency head coil. The participant's head was placed at the iso-center of the scanner with two foam pads either side to minimize head movements. Following an initial survey image, a Magnetisation

Prepared RApid Gradient Echo (MP-RAGE) anatomical image of the whole brain (120 slices, $1mm^3$ voxel size, TR of 7.3ms) was obtained to aid the placement of ROIs for spectroscopy and to allow for tissue segmentation to permit the measurement of tissue content within each voxel.

To further aid localisation of the hand area of the primary motor cortex (M1) and the Supplementary Motor Area (SMA), participants were asked to perform a brief bimanual, sequential, finger-thumb opposition task (i.e., for both hands to continuously tap the thumb against each finger sequentially until instructed to stop) while functional MR images (EPI sequence with a repetition time (TR) of 2s, echo time (TE) of 25ms, 2x2x3mm voxel size and a field of view of 192 x 60 x 192mm) were collected. 20 slices were positioned so as to capture activation within motor cortex, SMA and visual cortex, but not sub-cortical structures. Participants wore prism glasses in the scanner that allowed them to see a screen upon which visual stimuli were projected. Stimuli consisted of the word TAP written in red capital letters displayed for 24 seconds, followed by a white fixation dash (-) on a black background for 36 seconds. This sequence of visual stimuli was controlled using Presentation software and was repeated twice. Significant regions of blood oxygenated level dependant (BOLD) signal activation for the tap > rest contrast were identified in real-time using the IViewBOLD software built in to the Philips MRI system.

MR Spectroscopy data were collected sequentially from three different brain regions: the Supplementary Motor Area (SMA), the left hand area of primary motor cortex (M1), and primary visual cortex (V1), using a cubic $20mm^3$ voxel for each area. The SMA voxel was positioned on the mid-sagittal plane anterior to the central sulcus, using the peak BOLD activation in this region as a guide. The left hemisphere M1 voxel was positioned by identifying the hand area's characteristic Ω shape of the central sulcus on the MPRAGE as an anatomical landmark. This region was then confirmed by consulting the statistical contrast map generated from the Tap > Rest fMRI contrast and identifying the peak BOLD activation within this region. The V1 voxel was positioned in primary visual cortex, within the posterior region of the occipital lobe, centred on the mid-sagittal plane so as to cover both hemispheres, and was located using the calcarine sulcus from the MPRAGE as a landmark.

MRS data were acquired using a Stimulated Echo Acquisition Mode (STEAM) sequence with echo time TE/TM/TR= 16/17/2000ms. The width of the acquired spectrum was 4,000Hz with 4096 time points. A B_0 field map and a parcellated shimming approach was used to increase the B_0 homogeneity [**S5**]. 288 spectra were collected individually with Multiply Optimized Insensitive Suppression Train (MOIST) water suppression [**S6**], and two spectra were acquired without water suppression for correction to absolute concentrations using water referencing. Each MRS voxel took approximately 10minutes to complete. During this period participants were asked to remain as still as possible. In this respect neurotransmitter concentrations were measured at rest but participants may have been actively suppressing movements, including the occurrence of tics in the case of the individuals with TS.

Data processing

For all individual MR spectra, data from each of the 32 coil elements were collected separately, and then realigned, phase corrected, and averaged before being combined across coils using the theoretically optimized S/N^2 weighting, as described by Hall et al. [**S7**] using a Matlab script (Mathworks inc. Natick, USA) developed in-house for this purpose.

Spectra were then analysed using LCModel (version 2.2-4, Provencher, 1993), taking the unsuppressed water signal as an internal reference for metabolite quantification and for eddy current correction to gain concentration estimates for metabolites of interest. The simulated spectra of 20 metabolites are in the LCModel basis dataset [**S8**], including: γ-amino-butyric acid (GABA), Glutamine (Gln), Glutamate (Glu), and N-acetylaspartate (NAA). Previously published chemical shifts and coupling constants with TE/TM values identical to those used for data acquisition were used to stimulate spectra for a STEAM sequence [**S9**]. All spectra were visually inspected for movement artefact. Any outputs with an S.D. >30 were excluded from the analysis.

Functional MRI data was processed using SPM8 (The Welcome Trust Centre for Neuroimaging, London, UK). Data was realigned and re-sliced but were kept in native space. A t-contrast map for the tap > rest contrast with a family-wise error (FWE)

corrected threshold of $p<0.05$ was applied for each subject. The area of peak blood-oxygenated-level-dependant (BOLD) signal in left hemisphere M1 and the SMA was identified in each case. A $10mm^3$ region of interest (ROI) box was centred on the co-ordinates for each of these peak-activations using the Marsbar toolbox [**S10**]. The estimated average BOLD signal change for the tap > rest contrast was then extracted from each ROI for each participant.

Anatomical MPRAGE images were analysed to estimate the fraction of cerebral spinal fluid (CSF), gray matter (GM) and white matter (WM) that made up each VOI. The brain tissue was extracted from the skull and segmented into WM, GM and CSF using FSL software (Analysis Group, FMRIB, Oxford, UK). Binary segmentation with a cut off of 0.15 was used so that each voxel was classified as belonging to only one class. These segmented images were then used to estimate the proportion of CSF, GM and WM in each ROI using an in-house built Matlab script. The CSF, GM and WM fractions were compared separately between groups using independent samples t-tests. There were no significant differences in the proportion of each tissue type between groups for each VOI (maximum $t(27)=1.2$, $p>0.1$). In the analyses reported absolute metabolite concentrations for each ROI were standardised for each individual participant using that individual's measured GM tissue fraction for that ROI.

Diffusion tensor imaging (DTI)

DTI data were obtained from all TS individuals participating in the ^{1}H-MRS study save for participants (TS013 and TS069). Diffusion data were obtained in a separate session on a different day using a 3 Tesla Phillips Achieva MRI scanner with a 32-channel SENSE head coil. Diffusion data were obtained using an EPI sequence consisting of 48 slices of 2x2x2mm voxels with a repetition time (TR) of 7803ms, 32 directions and a diffusion weighting (b-value) of 1000. The slices were positioned using an initial survey scan; centred on the midline and angled along the anterior commissure - posterior commissure (AC-PC) line identified on the mid-sagittal slice. A high-resolution T1-weighted anatomical image was collected in the same scan-session. The MPRAGE (Magnetization Prepared RApid Gradient Echo) scan consisted of 160 slices with 1x1x1mm voxel size, and a field of view of 240x160x224mm centred along the mid-plane

of the brain and angled to follow the AP-PC line. The TR was 8.26ms, with a total scan time of roughly 4minutes. Participants lay flat wearing ear plugs and ear defenders on to protect from scanner noise. Foam pads were placed either side of the participant's ears to restrict head movement and participants were asked to keep as still as possible for the duration of each scan.

DTI data Processing

DTI data was processed using the FDT toolkit in FSL. First, all scans were eddy-current corrected, then the brain was extracted from the skull using the BET brain extraction tool, with a fractional intensity threshold of 0.3. Diffusion maps were then computed, including fractional anisotropy (FA), mean diffusivity (MD), the 3 principal Eigen values ($\lambda_1, \lambda_2, \lambda_3$) and the 3 principle Eigen vectors (V_1, V_2, V_3). Diffusion maps were then transformed using nonlinear registration into 1x1x1mm standard MNI (Montreal Neurological Institute) space via a standard FA map template.

Tractography analyses

Brain Voyager QX 2.3 software was used to process DTI data for probablistic tractography. DTI data was co-registered to each subject's MPRAGE. Then fractional anisotropy (FA) and mean diffusivity (MD) maps were created in native space. The SMA was identified as the medial section of the gyrus anterior to the pre-central gyrus (the superior frontal gyrus). The SMA was marked as an ROI and fibres tracked from this region (see Figure 3A main text. Reproduced below for convenience as Figure S3). A $6mm^3$ ROI was then placed in the body of the corpus callosum where the fibres crossed hemisphere (Figure S3B). Fibres from this corpus callosum ROI were then tracked back up to the SMA to verify the accuracy of the tractography analyses (Figure S3C). Mean MD and FA values from the corpus callosum $6mm^3$ ROI were then extracted for each TS participant.

Supplemental References

S1. Behar KL, Rothman DL. In vivo NMR studies of glutamate–GABA–glutamine cycling in rodent and human cortex: the central role of glutamine. J Nutr 2001; 131: 2498–2504.

S2. Orth M, Munchau A, Rothwell JC. (2008). Corticospinal system excitability at rest is associated with tic severity in Tourette syndrome. Biological Psychiatry 64: 248–51.

S3. Draper A, Jude L, Jackson GM, Jackson SR. (in press). Motor excitability during movement preparation in Tourette syndrome. *Journal of Neuropsychology.*

S4. Leckman JF, Riddle MA, Hardin MT, Ort SI, Swartz KL, Stevenson J, Cohen DJ. (1989). The Yale Global Tic Severity Scale: initial testing of a clinician-rated scale of tic severity. J Am Acad Child Adolesc Psychiatry 28: 566-73.

S5. Poole M, Bowtell R. (2008). Volume parcellation for improved dynamic shimming. MAGMA, *21*, 31–40.

S6. Tarasöw E., Wiercin´ ska-Drapa"o A., Kubas B., Dzienis W., Orzechowska-Bobkiewicz A., Prokopowicz D., & Walecki J., (2003). Cerebral MR spectroscopy in neurologically asymptomatic HIV-infected patients. *Acta Radiologica, 44*, 206–212.

S7. Hall E.L., Stephenson M.C., Price D., & Morris P.G., (2013). Methodology for improved detection of low concentration metabolites in MRS: Optimised combination of signals from multi-element coil arrays. *Neuroimage, (in press).*

S8. Mekle R, Mlynarik V, Gambarota G, Hergt M, Krueger G, Gruetter R. (2009). MR spectroscopy of the human brain with enhanced singla intensity at ultrashort echo times on a clinical platform at 3T and 7T. *Magnetic Resonance in Medicine, 61*, 1279–1285.

S9. Govindaraju V., Young K., & Maudsley A.A. (2000). Proton NMR chemical shifts and coupling constants for brain metabolites. *NMR Biomed, 13*, 129–53.

S10. Matthew Brett, Jean-Luc Anton, Romain Valabregue, Jean-Baptiste Poline. Region of interest analysis using an SPM toolbox [abstract] Presented at the 8th International Conference on Functional Mapping of the Human Brain, June 2-6, 2002, Sendai, Japan. Available on CD-ROM in *NeuroImage* 16(2).
