# Supplementary material for: Increased GABA Contributes to Enhanced Control over Motor Excitability in Tourette Syndrome
Source: Curr Biol. 2014 Oct 6;24(19):2343–7. doi: 10.1016/j.cub.2014.08.038 (PMC4188813; doi:10.1016/j.cub.2014.08.038)
Supplement: Document S2. Article plus Supplemental Information [file mmc2.pdf]

Current Biology 24, 2343–2347, October 6, 2014 ©2014 The Authors http://dx.doi.org/10.1016/j.cub.2014.08.038

Report

Increased GABA Contributes to Enhanced Control over Motor Excitability in Tourette Syndrome

Amelia Draper,[1] Mary C. Stephenson,[2]
Georgina M. Jackson,[3] Sophia Pépés,[1] Paul S. Morgan,[4]
Peter G. Morris,[2] and Stephen R. Jackson[1,*]
[1]School of Psychology, University of Nottingham, Nottingham NG7 2RD, UK
[2]Sir Peter Mansfield Magnetic Resonance Centre, University of Nottingham, Nottingham NG7 2RD, UK
[3]Division of Psychiatry and Applied Psychology, Institute of Mental Health, School of Medicine, University of Nottingham, Nottingham NG7 2TU, UK
[4]Medical Physics and Clinical Engineering, Queen's Medical Centre, Nottingham NG7 2RD, UK

Summary

Tourette syndrome (TS) is a developmental neurological disorder characterized by vocal and motor tics [1] and associated with cortical-striatal-thalamic-cortical circuit dysfunction [2, 3], hyperexcitability within cortical motor areas [4], and altered intracortical inhibition [4–7]. TS often follows a developmental time course in which tics become increasingly more controlled during adolescence in many individuals [1], who exhibit enhanced control over their volitional movements [8–11]. Importantly, control over motor outputs appears to be brought about by a reduction in the gain of motor excitability [6, 7, 12, 13]. Here we present a neurochemical basis for a localized gain control mechanism. We used ultra-high-field (7 T) magnetic resonance spectroscopy to investigate in vivo concentrations of γ-aminobutyric acid (GABA) within primary and secondary motor areas of individuals with TS. We demonstrate that GABA concentrations within the supplementary motor area (SMA)—a region strongly associated with the genesis of motor tics in TS [14]—are paradoxically elevated in individuals with TS and inversely related to fMRI blood oxygen level-dependent activation. By contrast, GABA concentrations in control sites do not differ from those of a matched control group. Importantly, we also show that GABA concentrations within the SMA are inversely correlated with cortical excitability in primary motor cortex and are predicted by motor tic severity and white-matter microstructure (FA) within a region of the corpus callosum that projects to the SMA within each hemisphere. Based upon these findings, we propose that extrasynaptic GABA contributes to a form of control, based upon localized tonic inhibition within the SMA, that may lead to the suppression of tics.

Results

Tourette syndrome (TS) is associated with alterations in the development of brain networks that result in neural circuits with imbalanced excitatory and inhibitory influences [15]. It is generally acknowledged that cortical-striatal-thalamic-cortical (CSTC) circuits are dysfunctional in TS, with subsets of striatal neurons becoming active within inappropriate contexts, resulting in the disinhibition of thalamocortical circuits [3] and the hyperexcitability of motor regions of the brain [4–7] that in turn lead to the occurrence of tics [14].

TS has been linked to alterations in inhibitory γ-aminobutyric acid (GABA) signaling [15, 16]. Postmortem examination has demonstrated that there are substantial decreases in the number of GABA interneurons found within the striatum of individuals with TS [2], and positron emission tomography imaging has revealed widespread reductions in $GABA_A$ receptor binding in TS [17]. Finally, studies of cortical-spinal excitability (CSE) in TS have demonstrated reduced intracortical GABAergic inhibition [4–7]. Together, these findings predict reduced phasic GABAergic inhibition in individuals with TS, which has most often been interpreted as a primary cause of the disorder contributing to the occurrence of tics.

TS often follows a developmental time course characterized by a reduction in the frequency and intensity of tics during adolescence [1]. It has been proposed that individuals gain control over their tics through the development of compensatory mechanisms that lead to enhanced control over motor outputs based upon increased tonic inhibition [8, 10, 11, 18–20]. Consistent with this proposal, it has been shown that the gain of transcranial magnetic stimulation (TMS)-induced motor excitability (i.e., TMS recruitment curves) ([6]; see Supplemental Information available online) and the gain of motor excitability immediately prior to volitional movements are both significantly reduced in individuals with TS [7, 12, 13]. These findings have been interpreted as a secondary consequence of, or adaptation to, the disorder and have been associated with a reduction in clinical symptoms [6]. Importantly, it has been demonstrated that both reduced inhibition (e.g., reduced short-interval cortical inhibition) and enhanced inhibition (e.g., reduced gain for TMS-induced motor excitability) are observed in the same group of individuals with TS [6].

The supplementary motor area (SMA) is a likely focus for these control mechanisms. The SMA is a major site for thalamocortical projections [21] and has been linked previously to the volitional control of action [22] and nonconscious, effector-specific control of motor outputs [23]. GABA concentrations within the SMA are correlated with performance on behavioral tasks that index nonconscious control of motor outputs [24]. Most importantly, cortical excitability within the SMA is linked to the genesis of tics in TS. Thus, the hyperexcitability within primary motor cortex (M1) that is observed in TS is likely due to increased functional interaction between SMA and M1 [25]: there is increased activity in the SMA of individuals with TS that immediately precedes the occurrence of tics [14], and inhibitory repetitive TMS (rTMS) delivered to the SMA has been shown to decrease tic frequency in individuals with TS [26–28]. Here we offer a novel perspective on how this increased control over motor outputs can arise as a consequence of localized increases in tonic inhibition based upon increased levels of nonsynaptic, extracellular GABA concentration that operate to alter the gain of cortical spinal excitability (CSE) locally.

We used ^1H magnetic resonance spectroscopy (MRS) at ultra-high field (7 T) to investigate in vivo concentrations of

*Correspondence: stephen.jackson@nottingham.ac.uk
This is an open access article under the CC BY license (http://creativecommons.org/licenses/by/3.0/).

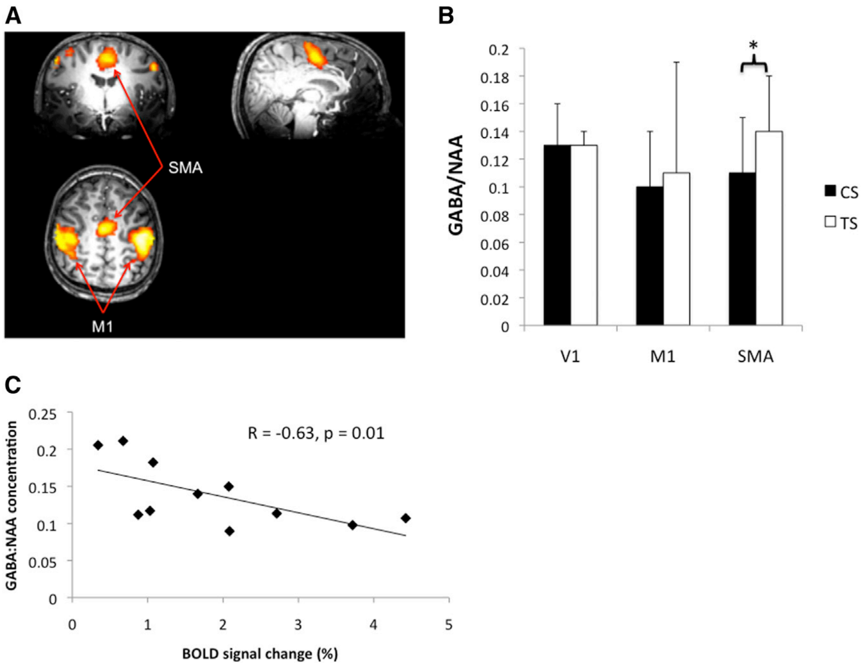

Figure 1. Results of Brain Imaging Analyses

(A) The fMRI BOLD signal associated with a bimanual sequential finger-thumb opposition task (tap > rest contrast) for a single representative participant that, for presentation purposes only, has been spatially smoothed.

(B) Mean GABA/NAA ratios for the Tourette syndrome (TS) and control group (CS) for each ROI. For the SMA ROI only, GABA concentrations are elevated relative to controls (*$p < 0.05$). Error bars are standard deviation.

(C) Scatterplot showing the negative association ($r = -0.63$, $p < 0.01$) between individual GABA/NAA ratios in the SMA voxel and fMRI BOLD signal change values within that same voxel.

GABA within the primary and secondary (SMA) motor areas of 15 adolescents (mean age 15.75 ± 3.05 years) with a confirmed clinical diagnosis of TS and a control group of age- and gender-matched typically developing individuals. MR spectroscopy data were collected from three 20 mm^3 regions of interest (ROIs) located within the hand area of left primary sensorimotor cortex (M1), and bilaterally from within the SMA and the primary visual cortex (V1). To aid localization of the hand area of M1 and SMA, participants performed a brief bimanual, sequential finger-thumb opposition task (i.e., with both hands continuously tap each finger sequentially against the thumb until instructed to stop) while functional MR images were obtained (Figure 1A).

Tissue Fraction Analyses

Anatomical (MP RAGE) images were analyzed to estimate, for each participant, the fraction of cerebral spinal fluid (CSF), gray matter (GM), and white matter (WM) within each volume of interest (VOI). The CSF, GM, and WM fractions were compared separately between groups using independent-samples t tests. These analyses confirmed that there were no significant differences in the proportion of each tissue type between groups for each VOI (maximum $t(27) = 1.2$, $p > 0.1$).

fMRI BOLD Analyses

Group differences in the magnitude of the fMRI blood oxygen level-dependent (BOLD) signal change for the tap > rest behavioral contrast were examined for each of the M1 and SMA VOIs using an independent-samples t test. The TS group had a significantly larger BOLD signal within the SMA VOI compared to controls (means: TS group = 1.88% ± 1.3%, control group = 1.1% ± 0.8%; $t(27) = 2.1$, $p < 0.05$). There was no significant between-group difference in fMRI BOLD within the M1 voxel.

GABA

The TS group had significantly increased absolute concentrations of GABA within the SMA compared to the control group (means: TS group = 1.1 ± 0.43 μmol/g, control group = 0.75 ± 0.28 μmol/g; $t(27) = 2.5$, $p < 0.05$). GABA concentrations within the M1 and V1 ROIs did not differ significantly between groups (both $p > 0.5$).

GABA/NAA Ratio

Following convention (e.g., [29]), we measured GABA concentrations as a ratio of *N*-acetylaspartate (NAA) concentrations within each ROI. Importantly, preliminary analyses confirmed that NAA concentrations did not differ significantly between groups for any of the three ROIs. The analyses revealed that GABA/NAA ratios were significantly increased relative to the matched control group within the SMA (means: TS group = 0.14 ± 0.04, control group = 0.11 ± 0.04; $t(27) = 2.2$, $p < 0.05$) but were not different from control group levels for the M1 or V1 ROIs (Figure 1B). (See Supplemental Information for identical findings for the GABA/creatine ratio.)

Our finding of selectively increased concentrations of GABA within the SMA in individuals with TS (hereafter MRS-GABA) is consistent with the proposal that MRS-GABA primarily measures extracellular GABA concentrations that have been linked to alterations in levels of tonic inhibition ([30]; see [31] for review). A discussion of the cellular basis for tonic inhibition is beyond the remit of this paper (but for recent reviews see [32–35]).

It should be noted that MRS-GABA was measured at rest in the current study, and participants were instructed to remain still throughout. This would require individuals with TS to actively suppress their tics. We cannot rule out that active suppression of tics contributed to the increased MRS-GABA in SMA that we observed. An increase in MRS-GABA in the SMA during tic suppression in TS would be consistent with previous reports that MRS-GABA concentrations within the SMA of neurologically healthy individuals correlate with individual levels of performance on behavioral tasks that index control of motor outputs [24].

Relationship between GABA Concentration and fMRI BOLD Response in TS

We investigated the association between MRS-GABA and fMRI BOLD within the SMA and M1 voxels. Previous studies reported a negative correlation between MRS-GABA concentrations and fMRI BOLD in the visual and motor cortices of healthy adults [29, 36, 37]. Pearson correlation coefficients were calculated for each group separately for each VOI. For

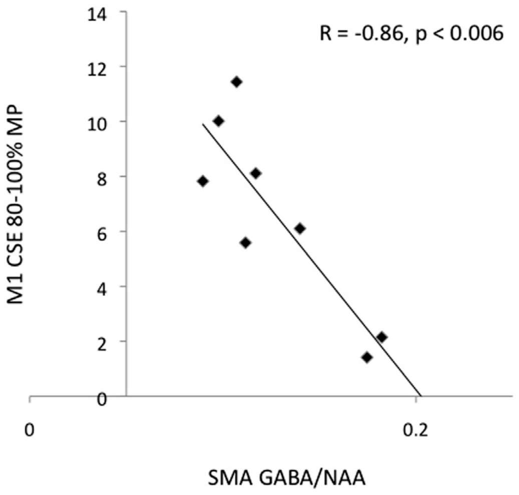

Figure 2. Association between SMA GABA Concentration and Motor Excitability

Individual levels of GABA concentration within the SMA ROI are inversely related to motor excitability immediately prior (81%–100%) to the execution of volitional movements. Motor excitability was measured by recording motor evoked potentials from the right hand following single-pulse TMS delivered to the left primary motor cortex. See Supplemental Information for further details.

the TS group, the analyses confirmed that the fMRI BOLD signal change within the SMA voxel was significantly negatively correlated with GABA/NAA ratio ($r = -0.65$, $p < 0.01$). Scatterplots showing these data are presented in Figure 1C. The correlation between fMRI BOLD response and GABA/NAA within the M1 VOI did not approach statistical significance ($p > 0.1$). The correlations between MRS-GABA and fMRI BOLD in M1 and SMA did not reach statistical significance for controls. Our finding that MRS-GABA concentrations within the SMA are inversely associated with the fMRI BOLD response is consistent with previous reports [29, 36, 37]. It is also consistent with the proposal that increases in MRS-GABA are linked to localized increases in tonic inhibition [30, 31], and that increased control over motor outputs in TS are brought about by reducing the gain of corticospinal excitability in cortical motor regions through increased tonic inhibition [7, 12, 13].

Relationship between GABA Concentration and Cortical-Spinal Excitability in TS

Individuals with TS exhibit significantly reduced gain for TMS-induced CSE [6] and also preceding the execution of volitional movements [7, 12, 13]. Furthermore, gain in CSE is inversely related to tic severity ([13]; see Supplemental Information). These findings have been interpreted as evidence that the gain of CSE is reduced in TS due to increased levels of tonic inhibition [7, 13].

To investigate the relationship between MRS-GABA and CSE in TS, we examined the Pearson correlation between MRS-GABA within SMA and levels of CSE within primary motor cortex (M1). CSE was measured using single-pulse TMS delivered to the hand area of the left M1 region in the period immediately preceding (81%–100% of the movement preparation period) volitional movements of the right hand in a subset of TS patients who had taken part in the current study and also in the study reported by Draper et al. [13]. This analysis revealed a significant negative correlation ($R = -0.86$, $p < 0.006$) between MRS-GABA in the SMA ROI and CSE measured within the left M1 (Figure 2). Although statistically significant, this result should be interpreted with caution due to the relatively small sample size. Nevertheless, taken together with the finding for fMRI BOLD reported above, it indicates that increases in MRS-GABA within the SMA in the TS group are likely associated with decreases in motor excitability.

If MRS primarily measures nonsynaptic, extracellular GABA concentrations that are linked to ambient levels of tonic inhibition [30, 31], then a key issue is to understand factors associated with MRS-GABA increases. One possibility is that MRS-GABA increases are triggered by neural projections arriving from other brain areas; another is that they are associated with tic severity scores.

Relationship between SMA GABA Concentration and WM Microstructure within the Corpus Callosum

As noted previously, the SMA is a major site for thalamocortical and cortical-cortical projections [21], and cortical excitability within the SMA is strongly linked to the genesis of tics in TS. Previous studies have demonstrated altered WM microstructure (i.e., reduced fractional anisotropy [FA] values in TS relative to matched controls) within regions of the corpus callosum (CC) linking sensorimotor areas of cortex. Furthermore, these alterations in FA are positively correlated with tic severity [20, 38].

We used diffusion tensor imaging (DTI) and tractography to test the hypothesis that increased projections to and from the SMA, as measured by FA values in the region of the CC projecting to the SMA, would be positively associated in TS with increased tic severity and increased MRS-GABA in the SMA. We identified a 6 mm^3 ROI within the body of the CC from which fibers clearly connected to the SMA within each hemisphere (Figure 3A; see Supplemental Information for details). We then measured mean FA values within this CC ROI for the TS group and correlated these with tic severity scores and MRS-GABA within the SMA ROI. The analyses revealed that FA values within the CC ROI were positively correlated with motor tic severity scores ($R = 0.87$, $p < 0.001$). These data confirm the previous finding that a reduction in CC projections to motor cortical areas is associated with reduced motor tic severity [20, 38]. More importantly, the analyses also revealed that FA within the CC ROI was significantly positively correlated with MRS-GABA within the SMA ($R = 0.75$, $p < 0.05$).

Linear Regression Analyses

To investigate which of several key variables might contribute to MRS-GABA concentrations within the SMA in the TS group, we carried out linear regression analyses for the following variables: participant age, current tic severity (Yale Global Tic Severity Scale [YGTSS] impairment, global, and motor scores), and WM microstructure (FA) in the region of the CC projecting to the SMA. The analyses confirmed that participant age and nonmotor indices of tic severity (i.e., YGTSS impairment and global scores) were not significant predictors of MRS-GABA within the SMA ($p > 0.05$). By contrast, motor tic severity (YGTSS motor score) (RSq = 0.54, Adj-Rsq = 0.46, $F = 6.97$, $p < 0.04$) and callosal FA (RSq = 0.62, Adj-Rsq = 0.55, $F = 9.7$, $p < 0.025$) were each significant predictors of MRS-GABA within the SMA for the TS group.

However, callosal FA is itself highly positively correlated with motor tic severity ($R = 0.87$, $p < 0.001$). To determine the

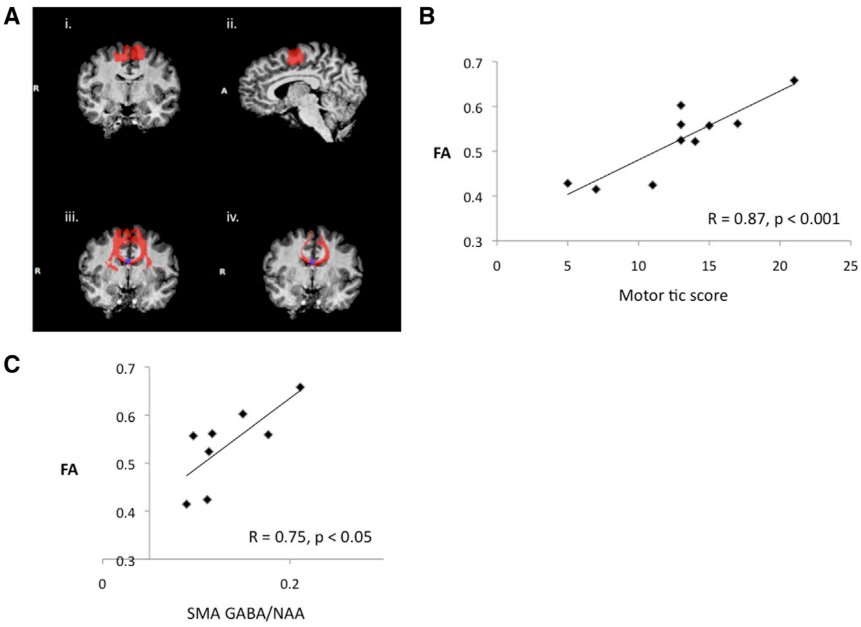

Figure 3. Association between SMA GABA Concentration and WM Microstructure

(Ai and Aii) An example ROI located in the SMA, displayed in the axial (Ai) and sagittal (Aii) planes.
(Aiii) Fibers tracked from the SMA region (red) and the location of the 6 mm^3 ROI located within the body of the corpus callosum where the fibers cross between the hemispheres (blue).
(Aiv) Confirmation that fibers tracked from the central 6 mm^3 ROI located within the corpus callosum terminate within the SMA.
(B) Scatterplot showing the significant positive association ($r = 0.87$, $p < 0.001$) between individual fractional anisotropy (FA) values within corpus callosum ROI and motor tic severity.
(C) Scatterplot showing the significant positive association ($r = 0.75$, $p < 0.05$) between individual FA values within corpus callosum ROI and GABA concentration values within the SMA voxel.

joint contribution of these factors, we entered them into a stepwise regression model. The analyses demonstrated that when motor tic severity is entered into the model first, callosal FA accounts for no additional variance and is no longer a significant predictor of MRS-GABA ($t = 1.13$, $p > 0.1$). By contrast, if callosal FA values are entered into the model first, motor tic severity accounts for no additional variance and is no longer a significant predictor of SMA GABA ($t = 0.42$, $p > 0.1$).

Discussion

We used ultra-high-field (7 T) ^{1}H MRS to investigate for the first time in vivo concentrations of GABA within primary and secondary motor areas of individuals with Tourette syndrome (TS). We demonstrate that concentrations of GABA within the SMA—a brain area consistently linked with the cortical genesis of motor tics in TS—are significantly elevated in individuals with TS relative to a control group of age-matched typically developing individuals. By contrast, GABA levels in primary motor cortex (M1) and in a control site within occipital cortex (V1) do not differ between groups.

We investigated the relationship between elevated MRS-GABA observed for the TS group and measures of motor tic severity, motor cortical excitability, fMRI BOLD response, and the structural connectivity of the SMA region. We demonstrate that MRS-GABA within the SMA is strongly negatively correlated with the fMRI BOLD response in SMA and also cortical excitability values within sensorimotor cortex, as measured by single-pulse TMS delivered immediately prior to a volitional movement of the contralateral hand [13]. Importantly, we report for the first time that MRS-GABA levels within the SMA are strongly positively predicted by both motor tic severity and the FA values within a region of the CC that projects to the SMA, and that these factors are themselves highly positively correlated.

It has been argued that an important secondary consequence of TS is that enhanced control over volitional movements, and the suppression of tics, may arise as a result of increased tonic inhibition [7–13]. This proposal is consistent with the repeated finding that the gain in cortical excitability is reduced in TS ahead of volitional movements [7, 12, 13] and in response to increasing levels of TMS stimulation [6]. Based upon the findings of the current study, we propose that this increase in tonic inhibition may be due to localized increases in extracellular GABA within the SMA. We believe that these findings are particularly important for understanding how localized adaptive changes in brain function may accompany neurodevelopmental disorders and play a key role in the control of behavioral symptoms.

Supplemental Information

Supplemental Information includes three figures, two tables, and Supplemental Experimental Procedures and can be found with this article online at http://dx.doi.org/10.1016/j.cub.2014.08.038.

Acknowledgments

Ethical committee approval for all procedures reported herein was obtained from the East Midlands branch of the National Research Ethics Service. This work was funded by MRC program grant G0901321 to P.G.M. and S.R.J. and a grant from the James Tudor Foundation to S.R.J. We are grateful to Jane Fowlie for assistance with participant recruitment.

Received: March 14, 2014
Revised: August 5, 2014
Accepted: August 18, 2014
Published: September 25, 2014

References

1. Leckman, J.F. (2002). Tourette's syndrome. Lancet *360*, 1577–1586.
2. Kalanithi, P.S., Zheng, W., Kataoka, Y., DiFiglia, M., Grantz, H., Saper, C.B., Schwartz, M.L., Leckman, J.F., and Vaccarino, F.M. (2005). Altered parvalbumin-positive neuron distribution in basal ganglia of individuals with Tourette syndrome. Proc. Natl. Acad. Sci. USA *102*, 13307–13312.
3. Albin, R.L., and Mink, J.W. (2006). Recent advances in Tourette syndrome research. Trends Neurosci. *29*, 175–182.
4. Orth, M., and Rothwell, J.C. (2009). Motor cortex excitability and comorbidity in Gilles de la Tourette syndrome. J. Neurol. Neurosurg. Psychiatry *80*, 29–34.
5. Gilbert, D.L., Bansal, A.S., Sethuraman, G., Sallee, F.R., Zhang, J., Lipps, T., and Wassermann, E.M. (2004). Association of cortical disinhibition with tic, ADHD, and OCD severity in Tourette syndrome. Mov. Disord. *19*, 416–425.

6. Orth, M., Münchau, A., and Rothwell, J.C. (2008). Corticospinal system excitability at rest is associated with tic severity in tourette syndrome. Biol. Psychiatry *64*, 248–251.
7. Heise, K.F., Steven, B., Liuzzi, G., Thomalla, G., Jonas, M., Müller-Vahl, K., Sauseng, P., Münchau, A., Gerloff, C., and Hummel, F.C. (2010). Altered modulation of intracortical excitability during movement preparation in Gilles de la Tourette syndrome. Brain *133*, 580–590.
8. Mueller, S.C., Jackson, G.M., Dhalla, R., Datsopoulos, S., and Hollis, C.P. (2006). Enhanced cognitive control in young people with Tourette's syndrome. Curr. Biol. *16*, 570–573.
9. Jackson, G.M., Mueller, S.C., Hambleton, K., and Hollis, C.P. (2007). Enhanced cognitive control in Tourette Syndrome during task uncertainty. Exp. Brain Res. *182*, 357–364.
10. Jung, J., Jackson, S.R., Parkinson, A., and Jackson, G.M. (2013). Cognitive control over motor output in Tourette syndrome. Neurosci. Biobehav. Rev. *37*, 1016–1025.
11. Jung, J., Jackson, S.R., Nam, K., Hollis, C., and Jackson, G.M. (2014). Enhanced saccadic control in young people with Tourette syndrome despite slowed pro-saccades. J. Neuropsychol. Published online April 16, 2014. http://dx.doi.org/10.1111/jnp.12044.
12. Jackson, S.R., Parkinson, A., Manfredi, V., Millon, G., Hollis, C., and Jackson, G.M. (2013). Motor excitability is reduced prior to voluntary movements in children and adolescents with Tourette syndrome. J. Neuropsychol. *7*, 29–44.
13. Draper, A., Jude, L., Jackson, G.M., and Jackson, S.R. (2013). Motor excitability during movement preparation in Tourette syndrome. J. Neuropsychol. Published online November 28, 2013. http://dx.doi.org/10.1111/jnp.12033.
14. Bohlhalter, S., Goldfine, A., Matteson, S., Garraux, G., Hanakawa, T., Kansaku, K., Wurzman, R., and Hallett, M. (2006). Neural correlates of tic generation in Tourette syndrome: an event-related functional MRI study. Brain *129*, 2029–2037.
15. Ramamoorthi, K., and Lin, Y. (2011). The contribution of GABAergic dysfunction to neurodevelopmental disorders. Trends Mol. Med. *17*, 452–462.
16. Clarke, R.A., Lee, S., and Eapen, V. (2012). Pathogenetic model for Tourette syndrome delineates overlap with related neurodevelopmental disorders including Autism. Transl. Psychiatr. *2*, e158.
17. Lerner, A., Bagic, A., Simmons, J.M., Mari, Z., Bonne, O., Xu, B., Kazuba, D., Herscovitch, P., Carson, R.E., Murphy, D.L., et al. (2012). Widespread abnormality of the γ-aminobutyric acid-ergic system in Tourette syndrome. Brain *135*, 1926–1936.
18. Plessen, K.J., Wentzel-Larsen, T., Hugdahl, K., Feineigle, P., Klein, J., Staib, L.H., Leckman, J.F., Bansal, R., and Peterson, B.S. (2004). Altered interhemispheric connectivity in individuals with Tourette's disorder. Am. J. Psychiatry *161*, 2028–2037.
19. Serrien, D.J., Orth, M., Evans, A.H., Lees, A.J., and Brown, P. (2005). Motor inhibition in patients with Gilles de la Tourette syndrome: functional activation patterns as revealed by EEG coherence. Brain *128*, 116–125.
20. Jackson, S.R., Parkinson, A., Jung, J., Ryan, S.E., Morgan, P.S., Hollis, C., and Jackson, G.M. (2011). Compensatory neural reorganization in Tourette syndrome. Curr. Biol. *21*, 580–585.
21. Akkal, D., Dum, R.P., and Strick, P.L. (2007). Supplementary motor area and presupplementary motor area: targets of basal ganglia and cerebellar output. J. Neurosci. *27*, 10659–10673.
22. Nachev, P., Kennard, C., and Husain, M. (2008). Functional role of the supplementary and pre-supplementary motor areas. Nat. Rev. Neurosci. *9*, 856–869.
23. Sumner, P., Nachev, P., Morris, P., Peters, A.M., Jackson, S.R., Kennard, C., and Husain, M. (2007). Human medial frontal cortex mediates unconscious inhibition of voluntary action. Neuron *54*, 697–711.
24. Boy, F., Evans, C.J., Edden, R.A., Singh, K.D., Husain, M., and Sumner, P. (2010). Individual differences in subconscious motor control predicted by GABA concentration in SMA. Curr. Biol. *20*, 1779–1785.
25. Franzkowiak, S., Pollok, B., Biermann-Ruben, K., Südmeyer, M., Paszek, J., Thomalla, G., Jonas, M., Orth, M., Münchau, A., and Schnitzler, A. (2012). Motor-cortical interaction in Gilles de la Tourette syndrome. PLoS ONE *7*, e27850.
26. Mantovani, A., Lisanby, S.H., Pieraccini, F., Ulivelli, M., Castrogiovanni, P., and Rossi, S. (2006). Repetitive transcranial magnetic stimulation (rTMS) in the treatment of obsessive-compulsive disorder (OCD) and Tourette's syndrome (TS). Int. J. Neuropsychopharmacol. *9*, 95–100.
27. Kwon, H.J., Lim, W.S., Lim, M.H., Lee, S.J., Hyun, J.K., Chae, J.H., and Paik, K.C. (2011). 1-Hz low frequency repetitive transcranial magnetic stimulation in children with Tourette's syndrome. Neurosci. Lett. *492*, 1–4.
28. Le, K., Liu, L., Sun, M., Hu, L., and Xiao, N. (2013). Transcranial magnetic stimulation at 1 Hertz improves clinical symptoms in children with Tourette syndrome for at least 6 months. J. Clin. Neurosci. *20*, 257–262.
29. Stagg, C.J., Bachtiar, V., and Johansen-Berg, H. (2011). The role of GABA in human motor learning. Curr. Biol. *21*, 480–484.
30. Stagg, C.J., Bestmann, S., Constantinescu, A.O., Moreno, L.M., Allman, C., Mekle, R., Woolrich, M., Near, J., Johansen-Berg, H., and Rothwell, J.C. (2011). Relationship between physiological measures of excitability and levels of glutamate and GABA in the human motor cortex. J. Physiol. *589*, 5845–5855.
31. Stagg, C.J. (2014). Magnetic Resonance Spectroscopy as a tool to study the role of GABA in motor-cortical plasticity. Neuroimage *86*, 19–27.
32. Farrant, M., and Nusser, Z. (2005). Variations on an inhibitory theme: phasic and tonic activation of $GABA_{(A)}$ receptors. Nat. Rev. Neurosci. *6*, 215–229.
33. Koch, U., and Magnusson, A.K. (2009). Unconventional GABA release: mechanisms and function. Curr. Opin. Neurobiol. *19*, 305–310.
34. Roth, F.C., and Draguhn, A. (2012). GABA metabolism and transport: effects on synaptic efficacy. Neural Plast. *2012*, 805830.
35. Héja, L., Nyitrai, G., Kékesi, O., Dobolyi, A., Szabó, P., Fiáth, R., Ulbert, I., Pál-Szenthe, B., Palkovits, M., and Kardos, J. (2012). Astrocytes convert network excitation to tonic inhibition of neurons. BMC Biol. *10*, 26.
36. Donahue, M.J., Near, J., Blicher, J.U., and Jezzard, P. (2010). Baseline GABA concentration and fMRI response. Neuroimage *53*, 392–398.
37. Muthukumaraswamy, S.D., Edden, R.A.E., Jones, D.K., Swettenham, J.B., and Singh, K.D. (2009). Resting GABA concentration predicts peak gamma frequency and fMRI amplitude in response to visual stimulation in humans. Proc. Natl. Acad. Sci. USA *106*, 8356–8361.
38. Plessen, K.J., Grüner, R., Lundervold, A., Hirsch, J.G., Xu, D., Bansal, R., Hammar, A., Lundervold, A.J., Wentzel-Larsen, T., Lie, S.A., et al. (2006). Reduced white matter connectivity in the corpus callosum of children with Tourette syndrome. J. Child Psychol. Psychiatry *47*, 1013–1022.

Current Biology, Volume *24*

Supplemental Information

Increased GABA Contributes to Enhanced Control over Motor Excitability in Tourette Syndrome

Amelia Draper, Mary C. Stephenson, Georgina M. Jackson, Sophia Pépés, Paul S. Morgan, Peter G. Morris, and Stephen R. Jackson

Supplemental Information

Supplemental Figures

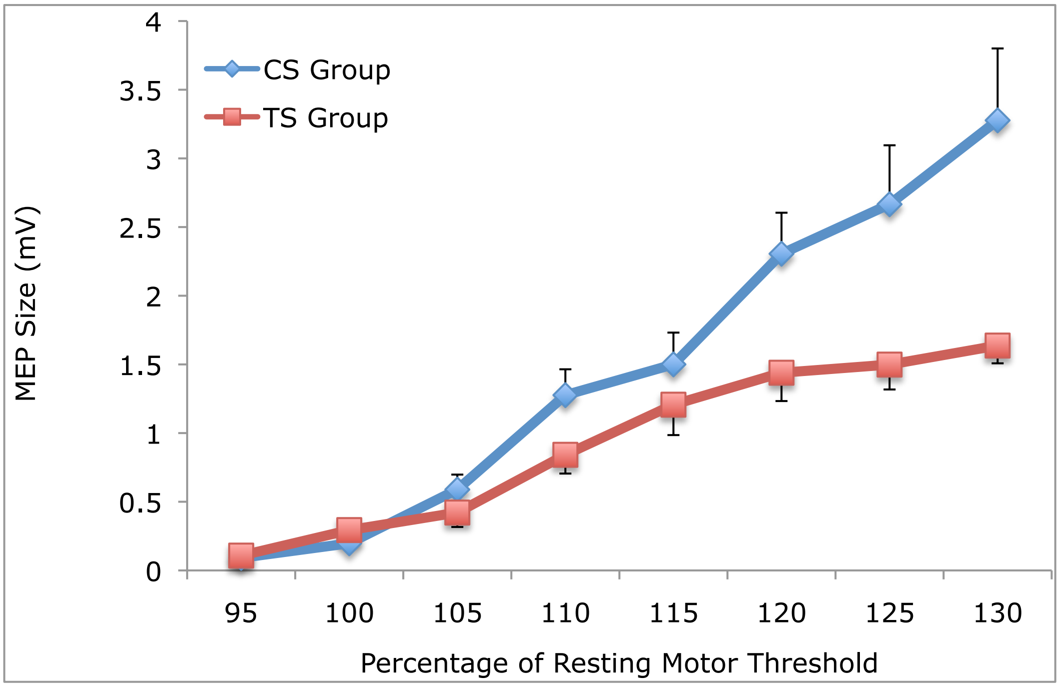

Figure S1: This figure relates to Figure 2 of the main text. It illustrates the recruitment curves for TMS-induced cortical spinal excitability for the TS group and controls. The graph shows the average MEP value for each group obtained from the FDI muscle of the right hand following single pulse TMS delivered to the left hand motor area at 95% - 130% of Resting Motor Threshold. Error bars are Standard Error.

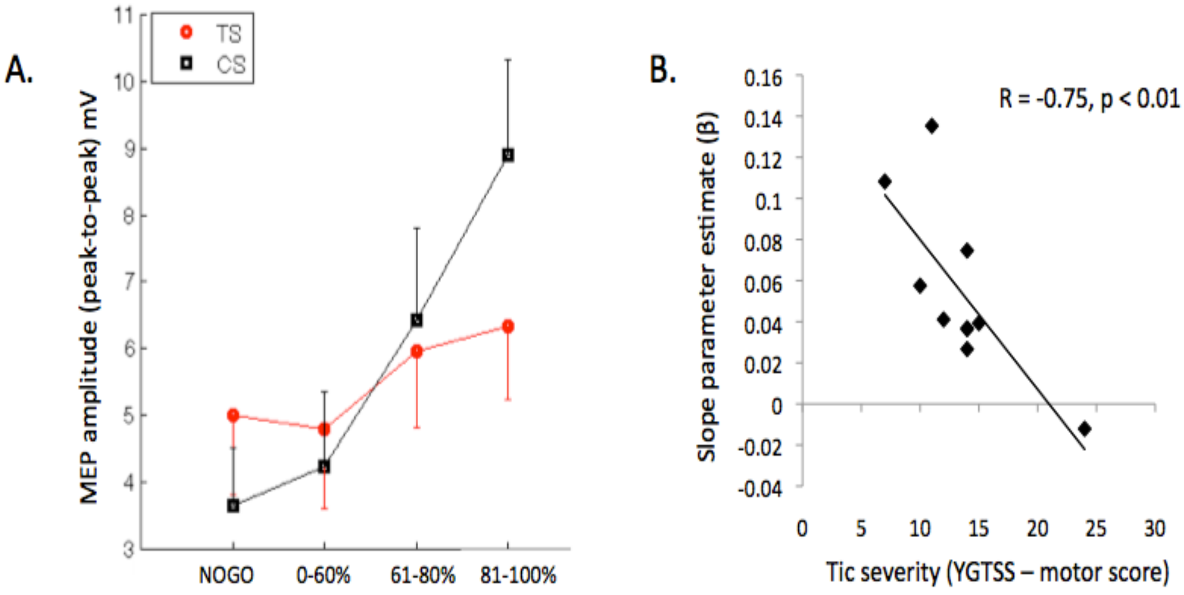

Figure S2: A. Data reproduced from Draper et al. [**S3**]. Illustrates differences between TS patients and matched controls in motor excitability immediately preceding the execution of volitional movements in a Go/NoGo task. The figure shows mean TMS-induced MEP amplitudes for TS and control groups when single pulse TMS was delivered on NoGo trials and 0-60%, 61-80%, or 81-100% of the movement preparation period on Go trials. Error bars are the standard error of the mean. **B.** Data reproduced from Draper et al. [**S3**] illustrating the relationship between motor tic increases in motor excitability, as indexed by the rise in MEP amplitude as a function of time through the movement preparation period.

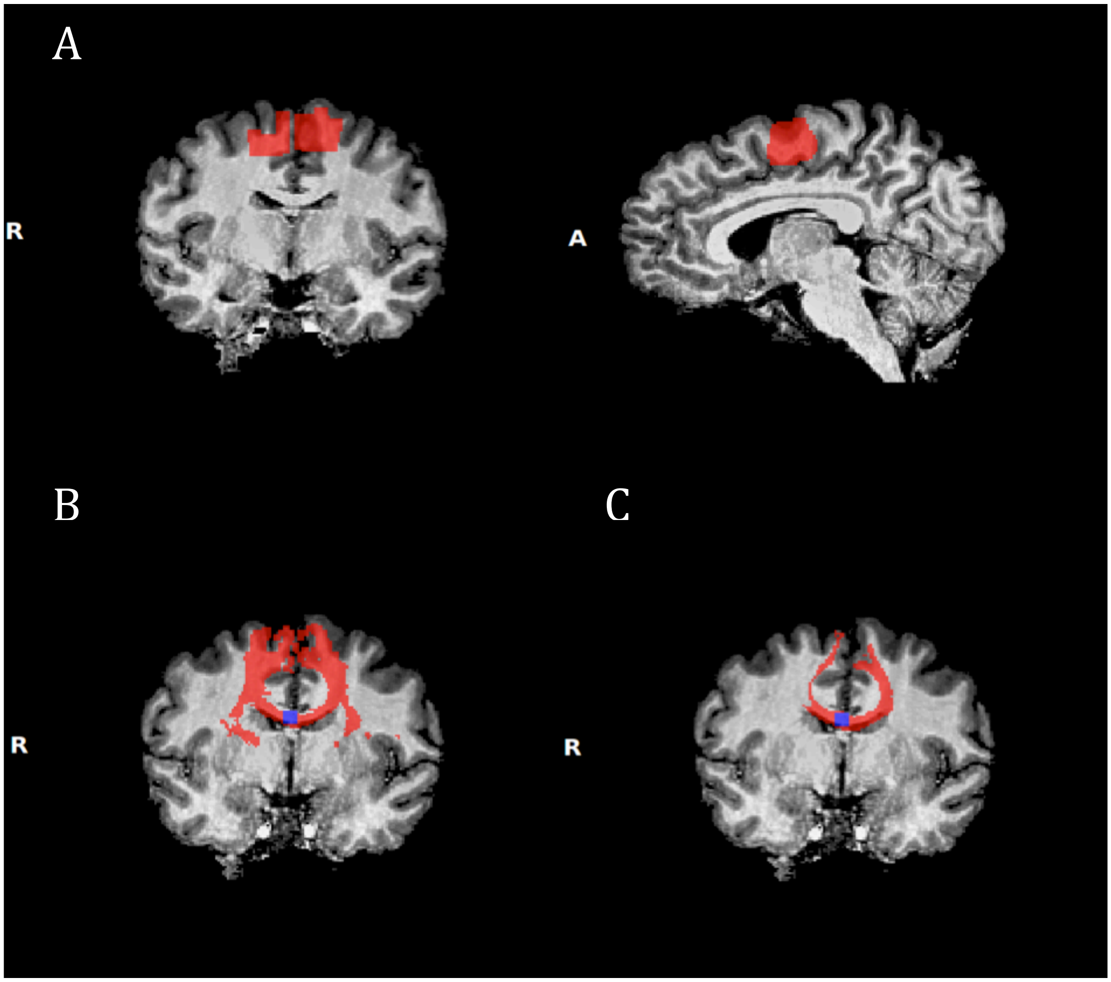

Figure S3: This figure relates to Figure 3 of the main text. A: Example of an ROI located in the supplementary motor area in the axial and sagittal plane. B: Illustrates fibres tracked from the SMA (red) and also the location of the 6mm^3 ROI positioned within the body of the corpus callosum where the fibres cross between the cerebral hemispheres (blue). C: Illustrates fibres that were tracked from the central 6mm^3 ROI terminate in the SMA.

Supplemental Tables

The focus of the current study was to investigate differences between the TS group and matched controls in MRS-GABA concentration within the primary and secondary motor areas, however for completeness we also report between group comparison of some other metabolites or metabolite ratios that may be of interest. These data are presented in Table S1 below.

N-acetylaspartate [NAA]

Absolute and tissue-corrected NAA concentration (i.e., absolute NAA values corrected for individual GM fractions) did not differ between the groups within any of the three ROIs.

GABA:NAA and GABA:Creatine (Cre) ratios

As reported in the main text, the GABA:NAA ratio was significantly increased for the TS group, but only within the SMA ROI. While we prefer to report the GABA:NAA ratio in the main text so as to maintain consistency with previous studies reporting GABA:NAA ratios, it is important to note that this finding is also observed if we examine for the GABA:Cr ratio (Table S1).

GABA/Glutamate (Glu) ratio

Effective cortical processing requires balanced excitatory and inhibitory influences. Glutamate is the main excitatory neurotransmitter within the human brain and GABA the main inhibitory neurotransmitter. As TS has been linked previously to imbalances in ambient levels of these neurotransmitters, we compared directly the GABA/Glu ratio for each group. The TS group had significantly higher GABA/Glu ratios in the SMA compared to those of the matched control group ($p<0.009$). By contrast, GABA/Glu ratios did not differ significantly between groups for either the M1 or V1 voxels (Table S1).

Glutamine (Gln)/Glu ratio

Within the brain the glutamine-glutamate cycle is key to the turnover and synthesis of both glutamate and GABA [**S1**]. Glutamate removed from the synaptic cleft is converted to glutamine within astrocytes. Astrocyte-derived glutamine is then used as a precursor for the synthesis of glutamate or GABA within neurons. For this reason, the Gln/Glu ratio is viewed as a potential marker for increases in brain activity. In the current study we found a marginal increase in the Gln/Glu ratio for the TS group within the SMA ROI ($p = 0.06$). The Gln/Glu ratio did not differ significantly between groups for either the M1 or V1 voxels.

**SMA ROI**			
**Metabolite**	**CS group**	**TS group**	**p-value**
*NAA**	6.9 ±0.47	7.3 ±1.27	NS
NAA	16.4 ±8.4	16.3 ±7.6	NS
GABA:NAA	0.11 ±0.04	0.16 ±0.06	**0.007**
GABA:Cr	0.26 ±0.14	0.34 ±0.14	**0.04**
GABA:Glu	0.10 ±0.04	0.13 ±0.04	**0.009**
Gln:Glu	0.23 ±0.07	0.26 ±0.08	0.06
**M1 ROI**			
**Metabolite**	**CS group**	**TS group**	**p-value**
*NAA**	8.0 ±0.82	7.3 ±1.25	NS
NAA	28.0 ±8.2	27.1 ±11.6	NS
GABA:NAA	0.10 ±0.04	0.11 ±0.08	NS
GABA:Cr	0.27 ±0.08	0.27 ±0.16	NS
GABA:Glu	0.11 ±0.04	0.10 ±0.05	NS
Gln:Glu	0.19 ±0.05	0.22 ±0.1	NS
**V1 ROI**			
**Metabolite**	**CS group**	**TS group**	**p-value**
*NAA**	8.9 ±0.8	8.4 ±0.8	NS
NAA	23.5 ±8.2	18.9 ±6.8	NS
GABA:NAA	0.13 ±0.03	0.13 ±0.06	NS
GABA:Cr	0.35 ±0.12	0.40 ±0.19	NS
GABA:Glu	0.13 ±0.03	0.14 ±0.06	NS
Gln:Glu	0.23 ±0.06	0.22 ±0.10	NS

Table S1: Comparison of additional metabolite concentrations and metabolite ratios for each group and region-of-interest (ROI). Absolute metabolite concentrations were standardised for each participant using that individual's measured GM fraction for each ROI. For completeness, and to aid comparison with previous studies, uncorrected concentrations of NAA are also presented (NAA*).

ID	Age (years)	Gender	IQ (WASI)	YGSS	Motor tic scores	Phonic tic scores	Co-morbitity	Medication
TS006	20.0	M	84	50	14	16	-	Clonidine
TS018	18.2	M	120	41	13	5	ADD	Clonidine
TS028	17.8	F	96	41	10	15	OCD	-
TS071	12.8	M	118	83	22	11	OCD	-
TS013	17.2	M	135	47	11	6	OCD	-
TS043	11.5	M	123	67	17	10	-	Clonidine
TS069	11.7	M	133	20	10	0	-	-
TS034	13.8	M	118	No tics last 2 months			-	-
TS062	17.0	M	131	65	24	21	-	Citalopram
TS048	14.8	M	118	28	12	6	-	Clonidine
TS049	20.2	M	116	64	21	13	-	Kapra
TS007	19.7	M	95	25	5	0	-	Clonidine
TS030	15.0	M	103	61	16	15	-	-
TS076	14.2	M	85	40	13	8	ADHD	Methylphenidate, Melatonin
TS074	12.4	M	102	30	10	8	-	Risperidone

Table S2: Clinical characteristics of subjects with Tourette's Syndrome (note: YGSS = Yale Global Tic Severity Scale; WASI= Wechsler Abbreviated Scale of Intelligence: vocabulary and matrix reasoning subtests; ADD = Attention Deficit Disorder; OCD = Obsessive-Compulsive Disorder; ADHD = Attention Deficit Hyperactivity Disorder).

Supplemental Experimental Procedures

1. Investigation of TMS-induced modulation of cortical-spinal excitability

Participants

Seventeen participants in total took part in this TMS study. Eight participants had Tourette syndrome, including 6 males, with a mean age of 18.3 ±2.7 years and a total Yale tic score ranging from 3 to 51. Nine controls were recruited that were gender and age matched to the TS group (7 males, mean age of 17.2 ±3.3 years).

Procedures

An unpaired Magstim Bistim 2 machine together with a 70mm figure-of-eight coil was used to deliver single-pulse TMS to the motor hotspot of the first dorsal interroseous (FDI) muscle in the right hand.

The motor hotspot was determined by a trial-and-error procedure. Once it had been found the resting motor threshold (RMT) was estimated at the motor hotspot based upon the stimulator intensity required to elicit an MEP of at least 150-200μV in 5 out of 10 trials. The location of this motor hotspot was subsequently tracked throughout the experiment using BrainSight 2. Specifically, a BrainSight tracking device was attached to the participant's forehead and to the TMS coil. A camera and software that aligns specific points on the participant's head to a virtual head display, and automatic curvilinear registration, allowed the experimenter to ensure that the TMS coil was always placed over the motor hotspot.

Once the motor hotspot was located, the TMS coil was stabilised using a manfrotto arm. The coil was continuously observed by the experimenter and adjusted whilst the trials were delivered to ensure the coil was always correctly positioned over the target. In order to record the TMS-induced muscle response, disposable electromyography electrodes with a diameter of 5mm were placed on the FDI muscle in a standard belly-tendon configuration. BrainVision Recorder software was used to record responses to the TMS protocol.

Participants rested their chin on a chin-rest whilst receiving 80 trials of single pulse TMS delivered at different percentages of their RMT with 5 seconds in between each

pulse. Intensities were delivered from 95% - 130% of RMT in increments of 5%. Pulses were pseudo-randomised and organised into 8 blocks (i.e., each block contained one trial of each intensity in a random order). After each block the experimenter checked that the participant was tolerating the procedure well and could readjust the coil position as necessary.

Results

Recruitment curves for the TS and control groups are presented in Figure S1. Inspection of this figure clearly indicates that the gain in motor excitability, as measured by mean MEP from the FDI muscle following single pulse TMS delivered to the left hand motor area at 95% - 130% of RMT, is reduced in the TS group.

To investigate this effect we carried out a 2-way mixed ANOVA with the between-subject factor Group (CS vs. TS) and the within subject factor Stimulator output (95% - 130% RMT). This analysis revealed significant main effects of Group ($F(1,15) = 6.2$, $p = 0.025$) and Stimulator output ($F(7,105) = 44.6$, $p < 0.00001$), and a significant Group x Stimulator output interaction ($F(7,105) = 5.4$, $p < 0.0001$).

This result confirms previous reports [**S2**] that the gain of TMS-induced motor excitability is significantly reduced in TS.

2. Cortical-spinal excitability prior to volitional movement in TS

A study investigating alterations in motor cortical-spinal excitability (CSE) ahead of volitional movements in individuals with TS, that was reported by Draper et al., [**S3**], was conducted concurrently with the current study, and eight of the current participants participated in both studies. As the Draper et al. study [**S3**] used TMS techniques to measure directly CSE ahead of volitional movements, it provides additional, independent, converging, evidence with respect to the relationship between MRS-GABA and motor excitability, and thus supports the finding reported in the current study that individual MRS-GABA concentrations are inversely related to fMRI BOLD activations. Full methodological details of the Draper et al., study are provided in [**S3**] and are summarised below. Figure S2 illustrates the core findings from the Draper et al. study.

Transcranical Magnetic Stimulation

Single pulse TMS was delivered to the hand area of the left hemisphere motor cortex. The TMS coil was positioned over the motor hotspot for the first dorsal interosseous (FDI) muscle of the right hand. Resting motor threshold (RMT) was estimated using an adapted staircase procedure and TMS pulse intensity was set to 110% of RMT throughout the experiment. Motor-evoked potentials (MEPs) were obtained from the FDI muscle of the right hand on each trial.

Experimental Procedure

Participants were seated with their head resting comfortably in a chin rest positioned 50cm away from a 17inch monitor that displayed the stimuli and completed a GO/NOGO motor task. On GO trials subjects pressed a response button with their right index finger whenever a 4cm diameter green filled circle, located centrally on a grey background, was presented to them. A red filled circle indicated a NOGO trial and subjects were instructed to withhold a response whenever the red circles appeared. Trials were presented in a pseudo-random order.

Participants completed 120 experimental trials and their median reaction time was re-calculated every 6 trials to control for any change in average speed of responding. A single TMS pulse was delivered on all trials at 25%, 50% or 75% of the individual's median reaction time relative to stimulus onset.

Electromyography (EMG) data was recorded on each trial for a period of 3 seconds following stimulus onset. EMG signals buffered and amplified using a g.USB Biosignal Amplifier (g-tech) with a sampling frequency of 1200Hz.

Statistical analyses

MEPs for all trials were visually inspected and any trials in which the MEP was ambiguous were excluded. TMS-induced MEPs were identified as the signal occurring directly after the characteristic TMS artefact, and MEP amplitude was defined as the peak-to-peak difference in amplitude measured in millivolts (mV). The *response-locked*

timing for when TMS pulse was delivered during the pre-movement period was calculated for each GO trial and expressed as the percentage of the reaction time for that trial, calculated as the interval separating stimulus onset time (0%) and response time (100%). Data were separated for each participant for TMS pulses delivered at 81-100% of *movement preparation* time. Median MEP amplitudes were then calculated for each individual.

3. ^{1}H Magnetic resonance Spectroscopy study

Participants

Fifteen adolescents (mean age = 15.75 ± 3.05 years, range = 11-20 years) with a confirmed clinical diagnosis of Tourette syndrome (TS) took part in this study. Participants were recruited from a specialised Tourette syndrome clinic at Queen's Medical Centre, Nottingham. Fourteen age and gender-matched (1 female, 13 males, mean age 15.86 ± 3.24 years) typically developing adolescents were recruited to act as a control group. Tic symptoms were measured on the day of testing using the Yale Global Tic Severity Scale (YGTSS) [**S4**]. Three TS participants had an additional diagnosis of co-morbid Obsessive Compulsive Disorder (OCD), one had an additional diagnosis of Attention Deficit Disorder (ADD) and one an additional diagnosis of Attention Deficit Hyperactivity Disorder (ADHD). Details of TS participants are presented in Table S2. For both groups IQ was estimated using the Wechsler Abbreviated Scale of Intelligence (WASI) using only the vocabulary and matrix reasoning subtests. Independent t-tests established that there were no significant differences between the groups in Age (TS group mean=15.75, CS group mean= 15.86, $t(27) = -0.1$, $p>0.1$) or IQ (TS group mean = 113, CS group mean = 120, $t(27) = 1.2$, $p>0.1$).

Magnetic Resonance Data Acquisition

Magnetic resonance data were acquired on a 7 Tesla Philips Achieva magnetic resonance imaging scanner with a 32-channel SENSE radio-frequency head coil. The participant's head was placed at the iso-center of the scanner with two foam pads either side to minimize head movements. Following an initial survey image, a Magnetisation

Prepared RApid Gradient Echo (MP-RAGE) anatomical image of the whole brain (120 slices, 1mm^3 voxel size, TR of 7.3ms) was obtained to aid the placement of ROIs for spectroscopy and to allow for tissue segmentation to permit the measurement of tissue content within each voxel.

To further aid localisation of the hand area of the primary motor cortex (M1) and the Supplementary Motor Area (SMA), participants were asked to perform a brief bimanual, sequential, finger-thumb opposition task (i.e., for both hands to continuously tap the thumb against each finger sequentially until instructed to stop) while functional MR images (EPI sequence with a repetition time (TR) of 2s, echo time (TE) of 25ms, 2x2x3mm voxel size and a field of view of 192 x 60 x 192mm) were collected. 20 slices were positioned so as to capture activation within motor cortex, SMA and visual cortex, but not sub-cortical structures. Participants wore prism glasses in the scanner that allowed them to see a screen upon which visual stimuli were projected. Stimuli consisted of the word TAP written in red capital letters displayed for 24 seconds, followed by a white fixation dash (-) on a black background for 36 seconds. This sequence of visual stimuli was controlled using Presentation software and was repeated twice. Significant regions of blood oxygenated level dependant (BOLD) signal activation for the tap > rest contrast were identified in real-time using the IViewBOLD software built in to the Philips MRI system.

MR Spectroscopy data were collected sequentially from three different brain regions: the Supplementary Motor Area (SMA), the left hand area of primary motor cortex (M1), and primary visual cortex (V1), using a cubic 20mm^3 voxel for each area. The SMA voxel was positioned on the mid-sagittal plane anterior to the central sulcus, using the peak BOLD activation in this region as a guide. The left hemisphere M1 voxel was positioned by identifying the hand area's characteristic Ω shape of the central sulcus on the MPRAGE as an anatomical landmark. This region was then confirmed by consulting the statistical contrast map generated from the Tap > Rest fMRI contrast and identifying the peak BOLD activation within this region. The V1 voxel was positioned in primary visual cortex, within the posterior region of the occipital lobe, centred on the mid-sagittal plane so as to cover both hemispheres, and was located using the calcarine sulcus from the MPRAGE as a landmark.

MRS data were acquired using a Stimulated Echo Acquisition Mode (STEAM) sequence with echo time TE/TM/TR= 16/17/2000ms. The width of the acquired spectrum was 4,000Hz with 4096 time points. A B_0 field map and a parcellated shimming approach was used to increase the B_0 homogeneity [**S5**]. 288 spectra were collected individually with Multiply Optimized Insensitive Suppression Train (MOIST) water suppression [**S6**], and two spectra were acquired without water suppression for correction to absolute concentrations using water referencing. Each MRS voxel took approximately 10minutes to complete. During this period participants were asked to remain as still as possible. In this respect neurotransmitter concentrations were measured at rest but participants may have been actively suppressing movements, including the occurrence of tics in the case of the individuals with TS.

Data processing

For all individual MR spectra, data from each of the 32 coil elements were collected separately, and then realigned, phase corrected, and averaged before being combined across coils using the theoretically optimized S/N^2 weighting, as described by Hall et al. [**S7**] using a Matlab script (Mathworks inc. Natick, USA) developed in-house for this purpose.

Spectra were then analysed using LCModel (version 2.2-4, Provencher, 1993), taking the unsuppressed water signal as an internal reference for metabolite quantification and for eddy current correction to gain concentration estimates for metabolites of interest. The simulated spectra of 20 metabolites are in the LCModel basis dataset [**S8**], including: γ-amino-butyric acid (GABA), Glutamine (Gln), Glutamate (Glu), and N-acetylaspartate (NAA). Previously published chemical shifts and coupling constants with TE/TM values identical to those used for data acquisition were used to stimulate spectra for a STEAM sequence [**S9**]. All spectra were visually inspected for movement artefact. Any outputs with an S.D. >30 were excluded from the analysis.

Functional MRI data was processed using SPM8 (The Welcome Trust Centre for Neuroimaging, London, UK). Data was realigned and re-sliced but were kept in native space. A t-contrast map for the tap > rest contrast with a family-wise error (FWE)

corrected threshold of $p<0.05$ was applied for each subject. The area of peak blood-oxygenated-level-dependant (BOLD) signal in left hemisphere M1 and the SMA was identified in each case. A 10mm^3 region of interest (ROI) box was centred on the co-ordinates for each of these peak-activations using the Marsbar toolbox [**S10**]. The estimated average BOLD signal change for the tap > rest contrast was then extracted from each ROI for each participant.

Anatomical MPRAGE images were analysed to estimate the fraction of cerebral spinal fluid (CSF), gray matter (GM) and white matter (WM) that made up each VOI. The brain tissue was extracted from the skull and segmented into WM, GM and CSF using FSL software (Analysis Group, FMRIB, Oxford, UK). Binary segmentation with a cut off of 0.15 was used so that each voxel was classified as belonging to only one class. These segmented images were then used to estimate the proportion of CSF, GM and WM in each ROI using an in-house built Matlab script. The CSF, GM and WM fractions were compared separately between groups using independent samples t-tests. There were no significant differences in the proportion of each tissue type between groups for each VOI (maximum $t(27)=1.2$, $p>0.1$). In the analyses reported absolute metabolite concentrations for each ROI were standardised for each individual participant using that individual's measured GM tissue fraction for that ROI.

Diffusion tensor imaging (DTI)

DTI data were obtained from all TS individuals participating in the ^{1}H-MRS study save for participants (TS013 and TS069). Diffusion data were obtained in a separate session on a different day using a 3 Tesla Phillips Achieva MRI scanner with a 32-channel SENSE head coil. Diffusion data were obtained using an EPI sequence consisting of 48 slices of 2x2x2mm voxels with a repetition time (TR) of 7803ms, 32 directions and a diffusion weighting (b-value) of 1000. The slices were positioned using an initial survey scan; centred on the midline and angled along the anterior commissure - posterior commissure (AC-PC) line identified on the mid-sagittal slice. A high-resolution T1-weighted anatomical image was collected in the same scan-session. The MPRAGE (Magnetization Prepared RApid Gradient Echo) scan consisted of 160 slices with 1x1x1mm voxel size, and a field of view of 240x160x224mm centred along the mid-plane

of the brain and angled to follow the AP-PC line. The TR was 8.26ms, with a total scan time of roughly 4minutes. Participants lay flat wearing ear plugs and ear defenders on to protect from scanner noise. Foam pads were placed either side of the participant's ears to restrict head movement and participants were asked to keep as still as possible for the duration of each scan.

DTI data Processing

DTI data was processed using the FDT toolkit in FSL. First, all scans were eddy-current corrected, then the brain was extracted from the skull using the BET brain extraction tool, with a fractional intensity threshold of 0.3. Diffusion maps were then computed, including fractional anisotropy (FA), mean diffusivity (MD), the 3 principal Eigen values ($\lambda_1, \lambda_2, \lambda_3$) and the 3 principle Eigen vectors (V_1, V_2, V_3). Diffusion maps were then transformed using nonlinear registration into 1x1x1mm standard MNI (Montreal Neurological Institute) space via a standard FA map template.

Tractography analyses

Brain Voyager QX 2.3 software was used to process DTI data for probablistic tractography. DTI data was co-registered to each subject's MPRAGE. Then fractional anisotropy (FA) and mean diffusivity (MD) maps were created in native space. The SMA was identified as the medial section of the gyrus anterior to the pre-central gyrus (the superior frontal gyrus). The SMA was marked as an ROI and fibres tracked from this region (see Figure 3A main text. Reproduced below for convenience as Figure S3). A $6mm^3$ ROI was then placed in the body of the corpus callosum where the fibres crossed hemisphere (Figure S3B). Fibres from this corpus callosum ROI were then tracked back up to the SMA to verify the accuracy of the tractography analyses (Figure S3C). Mean MD and FA values from the corpus callosum $6mm^3$ ROI were then extracted for each TS participant.

Supplemental References

S1. Behar KL, Rothman DL. In vivo NMR studies of glutamate–GABA–glutamine cycling in rodent and human cortex: the central role of glutamine. J Nutr 2001; 131: 2498–2504.

S2. Orth M, Munchau A, Rothwell JC. (2008). Corticospinal system excitability at rest is associated with tic severity in Tourette syndrome. Biological Psychiatry 64: 248–51.

S3. Draper A, Jude L, Jackson GM, Jackson SR. (in press). Motor excitability during movement preparation in Tourette syndrome. *Journal of Neuropsychology.*

S4. Leckman JF, Riddle MA, Hardin MT, Ort SI, Swartz KL, Stevenson J, Cohen DJ. (1989). The Yale Global Tic Severity Scale: initial testing of a clinician-rated scale of tic severity. J Am Acad Child Adolesc Psychiatry 28: 566-73.

S5. Poole M, Bowtell R. (2008). Volume parcellation for improved dynamic shimming. MAGMA, *21*, 31–40.

S6. Tarasöw E., Wiercin´ ska-Drapa"o A., Kubas B., Dzienis W., Orzechowska-Bobkiewicz A., Prokopowicz D., & Walecki J., (2003). Cerebral MR spectroscopy in neurologically asymptomatic HIV-infected patients. *Acta Radiologica, 44*, 206–212.

S7. Hall E.L., Stephenson M.C., Price D., & Morris P.G., (2013). Methodology for improved detection of low concentration metabolites in MRS: Optimised combination of signals from multi-element coil arrays. *Neuroimage, (in press).*

S8. Mekle R, Mlynarik V, Gambarota G, Hergt M, Krueger G, Gruetter R. (2009). MR spectroscopy of the human brain with enhanced singla intensity at ultrashort echo times on a clinical platform at 3T and 7T. *Magnetic Resonance in Medicine, 61*, 1279–1285.

S9. Govindaraju V., Young K., & Maudsley A.A. (2000). Proton NMR chemical shifts and coupling constants for brain metabolites. *NMR Biomed, 13*, 129–53.

S10. Matthew Brett, Jean-Luc Anton, Romain Valabregue, Jean-Baptiste Poline. Region of interest analysis using an SPM toolbox [abstract] Presented at the 8th International Conference on Functional Mapping of the Human Brain, June 2-6, 2002, Sendai, Japan. Available on CD-ROM in *NeuroImage* 16(2).
